# Essential role for paxillin tyrosine phosphorylation in LPS-induced mitochondrial fission, ROS generation and lung endothelial barrier loss

**DOI:** 10.1038/s41598-021-97006-y

**Published:** 2021-09-02

**Authors:** Panfeng Fu, Yulia Epshtein, Ramaswamy Ramchandran, Joseph B. Mascarenhas, Anne E. Cress, Jeffrey Jacobson, Joe G. N. Garcia, Viswanathan Natarajan

**Affiliations:** 1 Department of Pharmacology, University of Illinois at Chicago, COMRB Room # 3137, 909, South Wolcott Avenue, Chicago, IL 60612 USA; 2 The Affiliated Hospital of Medical School, Medical School of Ningbo University, 247 Renmin Road, Ningbo, China; 3 Department of Medicine, University of Illinois at Chicago, Chicago, IL USA; 4 Departments of Cellular and Molecular Medicine, University of Arizona Health Sciences, Tucson, AZ USA; 5 Department of Medicine, College of Medicine, University of Arizona Health Sciences, Tucson, AZ USA

**Keywords:** Cell signalling, Cell biology, Cell adhesion, Adherens junctions

## Abstract

We have shown that both reactive oxygen species (ROS) and paxillin tyrosine phosphorylation regulate LPS-induced human lung endothelial permeability. Mitochondrial ROS (mtROS) is known to increase endothelial cell (EC) permeability which requires dynamic change in mitochondrial morphology, events that are likely to be regulated by paxillin. Here, we investigated the role of paxillin and its tyrosine phosphorylation in regulating LPS-induced mitochondrial dynamics, mtROS production and human lung microvascular EC (HLMVEC) dysfunction. LPS, in a time-dependent manner, induced higher levels of ROS generation in the mitochondria compared to cytoplasm or nucleus. Down-regulation of paxillin expression with siRNA or ecto-expression of paxillin Y31F or Y118F mutant plasmids attenuated LPS-induced mtROS in HLMVECs. Pre-treatment with MitoTEMPO, a scavenger of mtROS, attenuated LPS-induced mtROS, endothelial permeability and VE-cadherin phosphorylation. Further, LPS-induced mitochondrial fission in HLMVECs was attenuated by both a paxillin siRNA, and paxillin Y31F/Y118F mutant. LPS stimulated phosphorylation of dynamin-related protein (DRP1) at S616, which was also attenuated by paxillin siRNA, and paxillinY31/Y118 mutants. Inhibition of DRP1 phosphorylation by P110 attenuated LPS-induced mtROS and endothelial permeability. LPS challenge of HLMVECs enhanced interaction between paxillin, ERK, and DRP1, and inhibition of ERK1/2 activation with PD98059 blocked mitochondrial fission. Taken together, these results suggest a key role for paxillin tyrosine phosphorylation in LPS-induced mitochondrial fission, mtROS generation and EC barrier dysfunction.

## Introduction

Paxillin is a multi-functional, multi-domain focal adhesion adaptor protein that serves as a scaffold for recruiting and binding to structural and signaling molecules^1^. The N-terminus of paxillin consists of 5 LD domains that are protein recognition sites, and the C-terminus has four tandem LIM domains, which mediate protein–protein interactions^2^. Also, the N-terminal of paxillin contains several proline-rich SH3 binding motifs with serine/threonine and tyrosine phosphorylation sites^2^. Paxillin through its multiple domains binds to structural and signaling proteins and regulates cell adhesion, migration, morphology and neovascularization^3–7^. The multiple serine/threonine and tyrosine phosphorylation sites in the paxillin are targeted by a diverse array of kinases that are activated in response to various adhesion stimuli, cytokines and growth factors^1,8^. Phosphorylation of tyrosine residues at the proline-rich SH3 motifs creates new binding sites for SH2-domain containing proteins, while serine/threonine phosphorylation in LIM domains potentiates the localization of paxillin to adhesions^9^. Thus, phosphorylation of paxillin regulates its localization and function as an adaptor molecule.

Three major paxillin N-terminal tyrosine phosphorylation sites, Y31 and Y118, and Y181 have been identified with Y31 and Y118 phosphorylation modulating docking of SH2 domain-containing proteins such as Crk, an adaptor molecule important for regulation of cell motility^8,10^. While Src and FAK are two prominent tyrosine kinases that phosphorylate paxillin at Y31 and Y118^11,12^, LPS-induced phosphorylation of paxillin at Y31 and Y118 is mediated by c-Abl and not by Src or FAK in human lung microvascular endothelial cells (HLMVECs)^13^. Further, c-Abl mediated tyrosine phosphorylation of Y31 and Y118 regulates LPS-induced pulmonary vascular permeability and injury^13^, and HGF- and S1P-induced reactive oxygen species (ROS) generation, lamellipodia formation and endothelial permeability^14^.

LPS is a bacterial endotoxin that is extensively used to mimic sepsis-induced injury both in vivo and in vitro. LPS induces secretion of pro-inflammatory cytokines, expression of adhesion molecules, endothelial permeability changes, and apoptosis via TLR2 and TLR4 signaling^15,16^, and stimulates ROS production in macrophages and lung endothelial cells^17^. ROS is known to play a role in LPS-induced endothelial permeability changes^17–19^ and apoptosis^20^. In mammalian cells LPS-mediated ROS is mainly generated by NADPH Oxidase (NOX) family proteins, and/or through mitochondrial electron transport^21–23^. Our previous study showed that c-Abl mediated paxillin tyrosine phosphorylation at Y31 and Y118 regulates LPS-induced endothelial dysfunction and lung injury^13^; however, the underlying molecular mechanism(s) by which paxillin regulates mitochondria (mt)-derived ROS dependent endothelial dysfunction is unknown.

Here, using HLMVECs as a model system, we have investigated the role of paxillin and paxillin Y31 and Y118 tyrosine phosphorylation in the regulation of LPS-mediated mtROS generation and endothelial dysfunction. Using ROS biosensors targeted to cytoplasm, mitochondria and nucleus, we show that LPS stimulation of HLMVECs resulted in increased mtROS compared to the cytoplasm or nucleus. Further, we delineated the pathway leading to LPS-mediated mtROS production via dynamin-related protein 1 (DRP1) activation and mitochondrial fission that was dependent on paxillin tyrosine phosphorylation at Y31 and Y118, and interaction between paxillin, DRP1 and ERK1/2. Blocking ERK1/2 activation by PD98059 attenuated mitochondrial fission and mtROS. Our results provide insight into the role of tyrosine phosphorylation of paxillin in regulating mtROS and endothelial barrier dysfunction via mitochondrial dynamics.

## Results

### LPS-stimulated mtROS production in human lung endothelial cells is attenuated by paxillin Y31F and Y118F mutants

**Figure 1:**
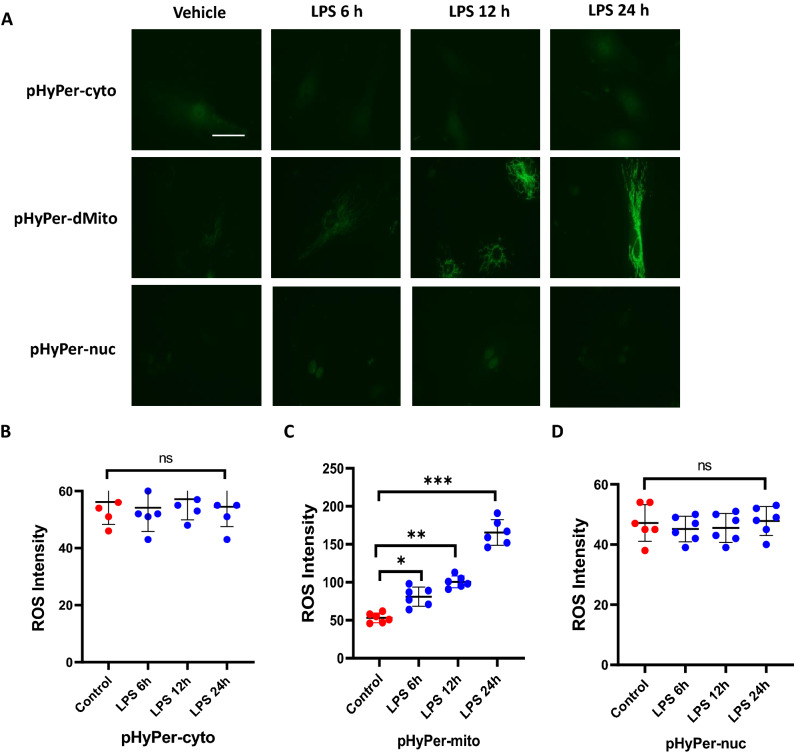
LPS induces mitochondrial ROS generation. (**A**) HLMVECs were transfected with ROS detector expressed in cytosol (pHyper Cyto), mitochondria (pHyper Mito), or nucleus (pHyper Nuclear). After 48 h of transfection, cells were treated with 100 ng/ml LPS or PBS for various time points. ROS images were acquired by fluorescent microscopy. Images were representative of 4–6 independent experiments. Scale bar: 5 μm. (**B**) Quantification of cytosol ROS, LPS did not induce significant ROS generation in cytosol. Data were presented as mean ± SD. (**C**) Quantification of mitochondrial ROS, data were presented as mean ± SD, *p < 0.05, LPS 6 h versus PBS control; **p < 0.01, LPS 12 h versus PBS control; ***p < 0.005, LPS 24 h versus PBS control. (**D**) Quantification of nuclear ROS, LPS did not induce significant nuclear ROS generation. Data were presented as mean ± SD.

**Figure 2:**
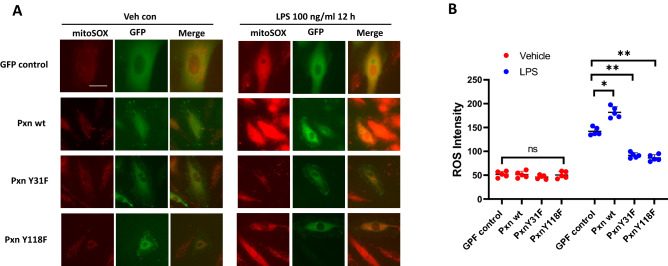
Paxillin mediates LPS-induced mitochondrial ROS. (**A**) HLMVECs were transfected with GFP-tagged control plasmid, wild type paxillin, paxillinY31F, or paxillin Y118F mutant plasmids before LPS treatment. Mitochondrial ROS were detected by MitoSOX dye. At least 20 GFP positive cells were selected for imaging. Images are representative of 5 independent experiments. Scale bar: 5 μm. (**B**) ROS were quantified by measurement of fluorescence intensity by Image J software. Data were presented as mean ± SD, *p < 0.05, LPS Pxn wt versus LPS control plasmid; **p < 0.01, LPS PxnY31F or LPS PxnY118 versus LPS control plasmid.

ROS generated in cells by NOX proteins and mitochondrial electron transport contributes to antimicrobial immunity^25–27^ and to intracellular signaling pathways^28,29^. LPS stimulates ROS production in macrophages and endothelial cells via Toll-like receptor 4 (TLR4) ligation with both, NOX and mitochondria involved in this process^30,31^. We have recently shown that paxillin and paxillin tyrosine phosphorylation stimulates p47^*phox*^-dependent ROS production by HGF and S1P in human lung ECs^14^. However, the role of mitochondria in LPS-induced ROS generation via paxillin in the endothelium is unclear. To determine the source of ROS after LPS challenge, HLMVECs were transfected with redox-sensitive biosensors that specifically target the cytoplasm, mitochondria, or the nucleus (pHyPer-cytosol, pHyPer-dMito or p-HyPer-nuc, respectively). The validity of pHyPer ROS assay was verified by addition of exogenous hydrogen peroxide (H_2_O_2)_ (5 µM) to HLMVECs transfected with pHyPer-cyto, pHyPer-mito or pHyPer-nuc for 1 h. Addition of exogenous hydrogen peroxide stimulated intensity of the biosensor fluorescence (pHyPer-cyto-control: 48 ± 15 vs. H_2_O_2_ 294 ± 38; pHyPer-mito-control: 54 ± 18 vs. H_2_O_2_ 168 ± 28; pHyPer-nuc: control: 42 ± 14 vs. H_2_O_2_ 80 ± 26). As shown in Fig. 1A–D, LPS challenge of HLMVECs increased, in a time-dependent manner (6–24 h), mtROS compared to cytoplasmic or nuclear ROS. Time points earlier to 6 h were not examined. These results with exogenous H_2_O_2_ show the functionality of the three pHyPer biosensors in determining LPS-induced ROS generation. Next, we investigated the role of paxillin tyrosine phosphorylation on LPS-mediated mtROS generation by over-expressing GFP-tagged wild type and paxillin Y31F and Y118F mutants in HLMVECs. LPS-increased mtROS was attenuated by overexpression of paxillin mutants Y31F and Y118F (Fig. 2A,B) with ecto-expression of wild type paxillin potentiating mtROS generation compared to non-transfected wild type cells. These data show that LPS-mediated ROS generation between 6 and 24 h is predominantly of mitochondrial origin in HLMVECs. However, the contribution of NOX enzymes in ROS generation earlier to 6 h in the cytoplasm is not ruled out.

### Paxillin mutants Y31F and Y118F block LPS-induced mitochondrial fragmentation in lung endothelial cells

**Figure 3:**
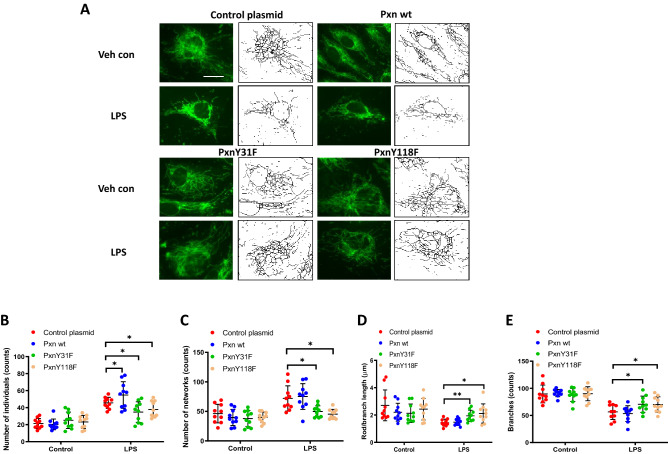
Paxillin mediates LPS-induced HLMVECs mitochondrial fission. (**A**) HLMVECs were transfected with Pxn wild type, PxnY31F, or PxnY118F plasmids. After 48 h of transfection, cells were stained with MitoTracker (50 nM, 10 min) before termination of LPS challenge (100 ng/ml, 12 h). Images were acquired by fluorescent microscopy and subjected to skeletonization by Image J software for the purpose of quantification as described in the Methods. Scale bar: 5 μm. (**B**–**E**) Mitochondrial dynamics was assessed by four major parameters of mitochondrial morphology, namely, number of individual fragments, number of networks, rod/branch length, and branch counts. Data were presented as means ± SD, for number of individual fragments, *p < 0.05 compared with LPS control plasmids; for number of networks, *p < 0.05, compared with LPS control plasmid; for rod/branch length, *p < 0.05, **p < 0.01 compared with LPS control plasmid; for branch counts, *p < 0.05 compared with LPS control plasmid.

Recent studies have shown that LPS via TLR4 signaling alter mitochondrial dynamics (fusion vs. fission) in microglia^32^ and macrophages^33,34^ that result in modulation of inflammatory responses both in vivo and in vitro. LPS causes endothelial cell dysfunction and apoptosis; however, the role of paxillin in LPS-induced alteration of mitochondrial morphology in lung ECs is unclear. To characterize mitochondrial morphological changes induced by LPS, HLMVECs pre-treated with MitoTracker were challenged with LPS (100 ng/ml), and mitochondrial morphological changes were monitored by confocal microscopy. As shown in Fig. 3 A-E, cells challenged with LPS for 24 h exhibited characteristics of mitochondrial fission based on mitochondrial length/fragmented (1 µm), tubular (1–3 µm) and elongated (> 3 µm) phenotypes^35^. The total number of fragmented structures was increased in LPS challenged HLMVECs whereas mitochondria with elongated morphology were decreased. In addition, the average mitochondrial length was significantly reduced in LPS-treated HLMVECs. Expression of the paxillin Y31F and Y118F mutants blocked LPS-induced mitochondrial morphological changes from fusion to fission with increased elongated and branched mitochondria in HLMVECs. These results show that LPS-induced tyrosine phosphorylation of paxillin at Y31 and Y118 is essential for switching mitochondrial morphology from fusion to fission in HLMVECs.

### Paxillin Mutants Y31F and Y118F attenuate LPS-induced DRP1 phosphorylation in lung endothelial cells

**Figure 4:**
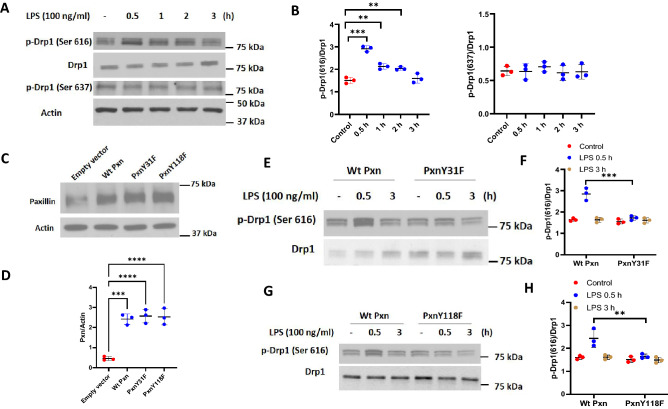
LPS-induced DRP1 phosphorylation requires both paxillin and ERK1/2. (**A**) HLMVECs were treated with LPS (100 ng/ml) for the indicated periods, phosphorylation of DRP1 at Ser616 and Ser637 was analyzed by Western blot. Equal protein loading was verified by probing with total actin and DRP1. (**B**) Results of quantitative analysis of (**A**) are shown as ratio of phosphorylated DRP1 to total DRP1. Values are means ± SD of 3 independent experiments. **p < 0.01, ***p < 0.005, compared with control. (**C**) HLMVECs were transfected with wild type paxillin, mutant paxillinY31F plasmid or mutant paxillinY118F. After 48 h of transfection, cells were lysed in cell lysis buffer, and cell lysates (20 µg protein) were subjected to SDS-PAGE and Western blotting with anti-paxillin antibody. Shown is a representative blot of three independent experiments. (**D**) The paxillin bands in (**C**) were quantified by image analysis. ***p < 0.005, compared to control cells transfected with empty plasmid backbone. ****p < 0.001, compared to control cells transfected with empty plasmid backbone. (**E**,**G**) HLMVECs were transfected with wild type paxillin, mutant paxillinY31F plasmid or mutant paxillinY118F. After 48 h of transfection, cells were treated with vehicle or LPS (100 ng/ml) for 0.5 and 3 h. Cell lysates (20 µgs protein) were subjected to SDS-PAGE and Western blotting with phospho-Ser616 DRP1 and anti-DRP1 antibodies. Shown is a representative blot of three independent experiments. (**F**,**H**) Results of quantitative analysis are shown as intensity ratio of phosphorylated DRP1 to total DRP1. Values are means ± SD of 3 independent experiments. ***p < 0.005, LPS 0.5 h wild type paxillin versus LPS 0.5 h mutant paxillinY31F. **p < 0.01, LPS 0.5 h wild type paxillin versus LPS 0.5 h mutant paxillinY118F.

Having demonstrated a role for paxillin Y31 and Y118 phosphorylation in LPS-mediated changes in mitochondrial morphology, we next determined if paxillin tyrosine phosphorylation is essential for DRP1 activation. DRP1 phosphorylation/dephosphorylation at Ser616 mediates mitochondrial fission^36,37^ while phosphorylation at Ser637 inhibits translocation of DRP1 to mitochondria^38^. LPS challenge of HLMVECs increased DRP1 phosphorylation at Ser616, but not Ser637, in a time-dependent manner without any change in total DRP1 protein expression (Fig. 4A,B). Transfection of HLMVECs with paxillin wild type and paxillin Y31F and Y118F mutants exhibited increased expression of the proteins compared to non-transfected cells (Fig. 4C,D). Interestingly, LPS-mediated DRP1 Ser616 phosphorylation at 30 min was attenuated by Y31F and Y118F mutants (Fig. 4E–H). Together, these results suggest a key role for paxillin Y31 and Y118 tyrosine phosphorylation in activation of DRP1 in lung ECs.

### Inhibition of DRP1 activation by P110 reduces LPS-induced mitochondrial fission and mtROS production in human lung endothelial cells

**Figure 5:**
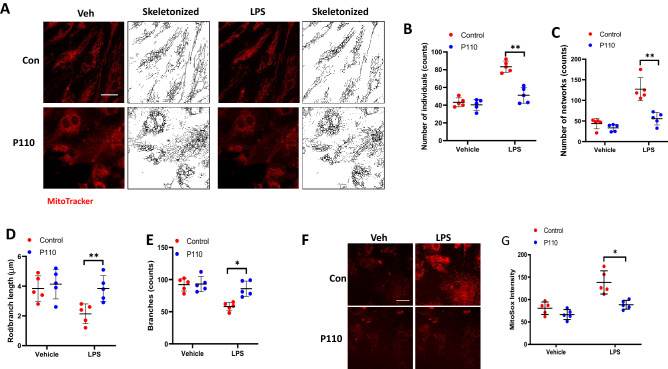
DRP1 inhibitory peptide blocks LPS-induced EC mitochondrial fission and ROS. (**A**) HLMVECs were pretreated with DRP1 inhibitory peptide P110 (1 µM) for 1 h followed by adding of MitoTracker 10 min before imaging. Images were acquired at 5 min intervals. After 30 min imaging of the basal level of mitochondrial morphological information, cells were treated with 100 ng/ml LPS followed by continuous imaging of mitochondria at the same intervals. Presents are images acquired before LPS treatment (Veh column) and after LPS treatment (LPS column). (**B**–**E**) Mitochondrial dynamics was assessed by number of individual fragments, number of networks, rod/branch length, and branch counts. Data were presented as means ± SD, for number of individual fragments, **p < 0.01, LPS alone versus LPS + P110; for number of networks, **p < 0.01, LPS alone versus LPS + P110; for rod/branch length, **p < 0.01, LPS alone versus LPS + P110; for branch counts, *p < 0.05, LPS alone versus LPS + P110. (**F**) HLMVECs were treated same as described above, instead of using MitoTracker staining, cells were treated with 5 µm MitoSOX for 10 min before imaging. Shown are representative of at least 5 images. Scale bar 10 μm. (**G**) MitoSOX intensity analysis, *p < 0.05, LPS alone versus LPS + P110.

To further determine the potential link between mitochondrial fission and mtROS production, we used a DRP1-specific peptide inhibitor P110^38,39^ in LPS-challenged lung endothelial cells. Pre-treatment of HLMVECs with P110 for 1 h followed by LPS challenge resulted in a significant reduction in fragmented mitochondria with a concomitant increase in tubular and elongated mitochondria, similar to control levels (Fig. 5A–E). Next, we investigated the relationship between mitochondrial fission and mtROS production after LPS challenge. HLMVECs were challenged with LPS for 24 h with or without P110 and then with MitoSOX for 1 h to quantify mtROS by confocal microscopy. As shown in Fig. 5F,G, inhibition of mitochondrial fission by P110 suppressed mitochondrial ROS generation. These results directly show that increased mitochondrial fission induced by LPS stimulates mtROS production in lung endothelial cells.

### ERK1/2 inhibition suppresses LPS-induced DRP1 phosphorylation, mitochondrial fission and mtROS production in lung endothelial cells

**Figure 6:**
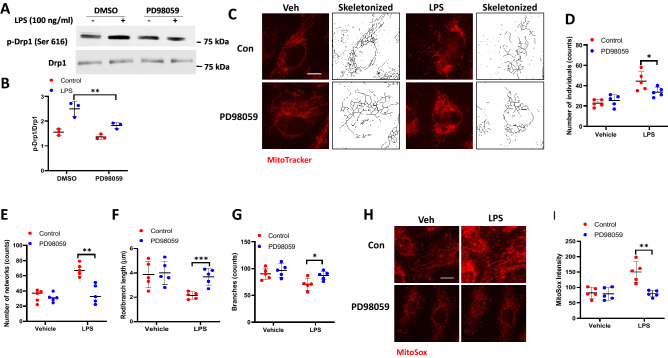
Erk mediates DRP1 phosphorylation and LPS-induced mitochondrial fission. (**A**) HLMVECs were pretreated with PD98059 for 30 min followed by LPS for 30 min, phosphorylation of DRP1 was analyzed by Western blot. (**B**) Results of quantitative analysis are shown as intensity ratio of phosphorylated DRP1 to total DRP1. Values are means ± SD of 3 independent experiments. **p < 0.01, LPS versus LPS + PD98059. (**C**) HLMVECs were pretreated with PD98059 for 30 min followed by adding of MitoTracker 10 min before imaging. (**D**–**G**) Mitochondrial dynamics was assessed by number of individual fragments, number of networks, rod/branch length, and branch counts. Data were presented as means ± SD, for number of individual fragments, *p < 0.05, LPS alone versus LPS + PD98059; for number of networks, **p < 0.01, LPS alone versus LPS + PD98059; for rod/branch length, ***p < 0.005, LPS alone versus LPS + PD98059; for branch counts, *p < 0.05, LPS along versus LPS + PD98059. Presents are representative of at least 5 images. Scale bar 5 μm. (**H**) HLMVECs were treated same as described above and staining with MitoSOX for 10 min before imaging. Shown are representative of at least 5 images. Scale bar 10 μm. (**I**) MitoSOX intensity analysis, *p < 0.05, LPS alone versus LPS + PD98059.

As ERK2-mediated DRP1 Ser616 phosphorylation drives mitochondrial fission^40,41^, we investigated the role of ERK1/2 in LPS-induced DRP1 phosphorylation, mitochondrial fission and mtROS production. Pre-treatment of HLMVECs with PD98059, an inhibitor of MEK1/2, reduced LPS-stimulated DRP1 Ser616 phosphorylation (Fig. 6A,B) and mitochondrial fission (Fig. 6C–G). Furthermore, PD98059 inhibited LPS-induced mtROS production in HLMVECs (Fig. 6H,I). These results show a role for ERK1/2 in LPS-mediated DRP1 phosphorylation, mitochondrial fission, and mtROS production in lung endothelial cells.

### Paxillin mutants attenuate LPS-stimulated association between paxillin, DRP1 and ERK1/2 in lung endothelial cells

**Figure 7:**
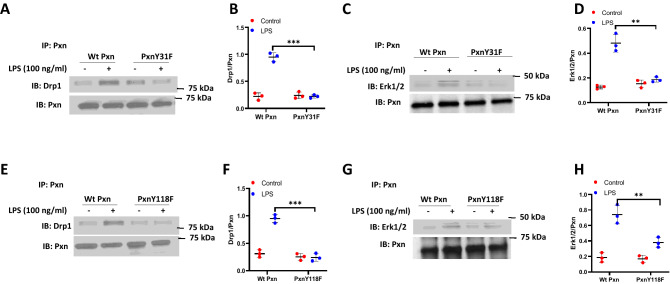
Phosphorylation of paxillin Y31 and Y118 is required for paxillin interactions with Erk1/2 and DRP1. (**A**–**D**) HLMVECs were transfected with wild type paxillin or paxillinY31F mutant plasmids, interactions of paxillin with DRP1 and Erk1/2 was assessed by IP experiment. Data were presented as means ± SD, **p < 0.01, LPS wild type paxillin versus LPS paxillinY31F mutant; ***p < 0.005, LPS wild type paxillin versus LPS paxillinY31F mutant, n = 3. E–H, HLMVECs were transfected with wild type paxillin or paxillinY118F mutant plasmids, interactions of paxillin with DRP1 and Erk1/2 was assessed by IP experiment. Data were presented as means ± SD, **p < 0.01, LPS wild type paxillin versus LPS paxillinY118F mutant; ***p < 0.005, LPS wild type paxillin versus LPS paxillinY181F mutant, n = 3.

To further understand how paxillin modulates LPS-induced ERK and DRP1 activation to regulate mitochondrial fission and mtROS, we investigated the potential interaction between paxillin, DRP1 and ERK1/2. HLMVECs transfected with vector control or paxillin Y31F and Y118F mutants were exposed to either vehicle or LPS, and cell lysates were subjected to immunoprecipitation with anti-paxillin antibody and analyzed for the presence of either DRP1 or ERK1/2. As shown Fig. 7A–H, paxillin is constitutively associated with DRP1, and ERK1/2 in control cells, with this association further increased by exposure to LPS (30 min). In contrast, the presence of the paxillin mutant proteins, Y31F and Y118F, suppressed the association of DRP1 and ERK1/2 in paxillin immunoprecipitates. These results suggest increased association between paxillin, DRP1 and ERK1/2 after LPS challenge of HLMVECs.

### Inhibition of DRP1 and mtROS production attenuates LPS-mediated VE-cadherin tyrosine phosphorylation and endothelial dysfunction

**Figure 8:**
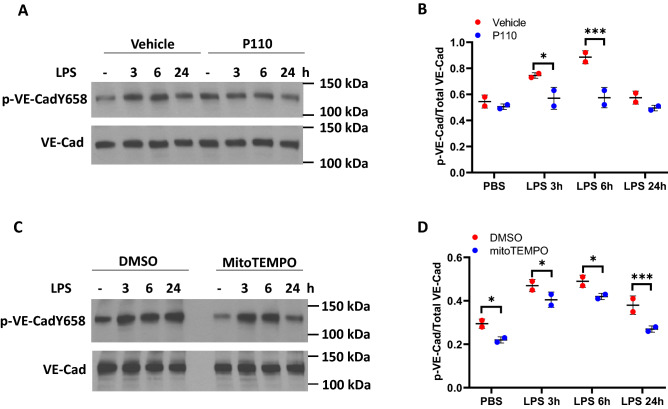
Inhibition of mitochondrial ROS dampens LPS-induced VE-Cadherin phosphorylation. HLMVECs were pretreated with P110 (**A**) or MitoTEMPO (**C**) to block mitochondrial ROS, cells were then treated with LPS for various time points. Phosphorylation of VE-Cadherin at Y658 was detected by Western blot. Two independent experiments were performed. (**B**,**D**) Results of quantitative analysis are shown as intensity ratio of phosphorylated VE-Cadherin to total VE-Cadherin. Values are means ± SD of 2 independent experiments. *p < 0.05, vehicle versus P110 for 3 h of LPS treatment (**B**) and vehicle versus MitoTEMPO for 3 h and 6 h (**D**) of LPS treatment; ***p < 0.005, vehicle versus P110 for 6 h of LPS treatment (**B**) and vehicle versus MitoTEMPO for 24 h of LPS treatment (**D**).

**Figure 9:**
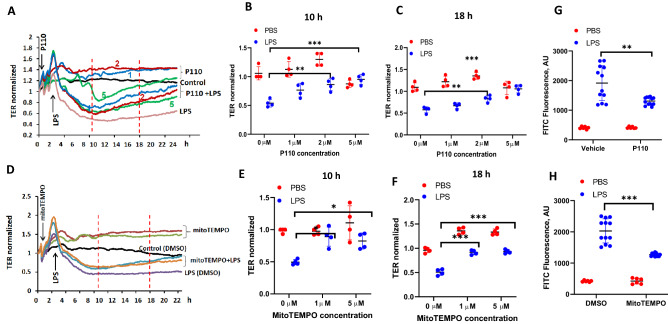
Inhibition of mitochondrial ROS attenuated LPS-induced HLMVEC barrier permeability. The effects of mitochondrial ROS on endothelial barrier function were tested by measurements of trans-endothelial electrical resistance (TER) (**A**–**F**) and FITC-dextran leakage (**G**,**H**). HLMVECs were pretreated with different concentrations of P110 (1–5 µM) or MitoTEMPO (1 or 5 µM) for 30 min followed by LPS (100 ng/ml) challenge. TER was recorded continuously for 24 h (**A**,**D**). Shown are representative tracings from 4 independent experiments. The TERs at 10 h and 18 h time points were used for statistical analysis (**B**,**C** & **E**,**F**). (**B**,**C**) Values are means ± SD. **p < 0.01, P110 2 μM versus LPS control, n = 4; ***p < 0.005, P110 5 μM versus LPS, n = 4. (**E**,**F**) Values are means ± SD. *p < 0.05, MitoTEMPO 1 or 5 μM versus LPS; ***p < 0.005, MitoTEMPO 1 or 5 μM versus LPS, n = 4. (**G**,**H**), HLMVECs monolayer permeability was assessed by FITC-dextran leakage as describe in METHODS. Values are means ± SD. **p < 0.01, P110 + LPS versus LPS, n = 4; ***p < 0.005, MitoTEMPO + LPS versus LPS, n = 4.

We have shown that c-Abl-mediated tyrosine phosphorylation of paxillin regulates LPS-induced endothelial dysfunction and lung injury^13^. While LPS-induced ROS also modulates endothelial cell permeability^19^, whether mitochondrial fission/mtROS play a role in LPS-induced endothelial dysfunction is unknown. As shown in Fig. 8 A-D, LPS-induced tyrosine phosphorylation of VE-cadherin at Y658 was suppressed by the DRP1 inhibitor, P110 and mtROS scavenger MitoTEMPO in HLMVECs. Next, we investigated the effect of P110 and MitoTEMPO on LPS-induced permeability determined by TER and FITC-dextran method. Addition of LPS (1 µg/ml) to HLMVECs reduced TER (increased permeability) starting at 4 h post LPS addition and decrease in TER was sustained up to 18 h, followed by a slight recovery (Fig. 9A,D). Pre-treatment of HLMVECs with P110 or MitoTEMPO significantly reduced LPS-induced barrier disruption as determined by restoration of TER (Fig. 9A–F) and inhibition of FITC-dextran leakage (Fig. 9G,H). These results suggest that LPS-induced barrier disruption is mediated by DRP1 activation and mtROS generation.

## Discussion

Paxillin plays an important role in cell morphology, migration, adhesion, and cell signal transduction. Our earlier studies defined a key role of paxillin and paxillin tyrosine phosphorylation at Y31 and Y118 in HGF/S1P-mediated ROS generation, lamellipodia formation and endothelial barrier function^14^. More recently, we showed that paxillin and paxillin tyrosine phosphorylation are implicated in LPS-induced endothelial permeability and lung injury^13^. Here, we now show a novel role for paxillin and paxillin tyrosine phosphorylation in LPS-induced mitochondrial fission and mtROS production. Further, we observed that blocking mitochondrial fission and mtROS reduced LPS-induced tyrosine phosphorylation of VE-cadherin and reversed endothelial dysfunction. These findings suggest that paxillin tyrosine phosphorylation regulates mitochondrial dynamics, and mtROS, which modulate LPS-induced lung endothelial permeability and barrier function.

As a multi-functional focal adhesion adaptor protein, paxillin exhibits LD motifs, LIM domains and SH2-binding sites that facilitate recruiting and binding to structural and signaling proteins^1^. These interactions play an important role in cell migration and adhesion and require tyrosine phosphorylation at Y31, Y118 and Y131 sites. Paxillin is tyrosine phosphorylated by FAK, the Src family of kinases and c-Abl at Y31 and Y118, which is critical for paxillin redistribution to focal adhesions and angiogenesis and endothelial barrier regulation^11,13^. Here we have demonstrated another novel role for paxillin in EC mitochondrial dynamics. The mitochondria are now recognized to form dynamic networks and undergo cycles between fusion and fission depending upon the external cues. The process of fusion is regulated by the transmembrane GTPases Mitofusin-1 and Mitofusin-2 and optic atrophy protein 1 (OPA1), while fission is mediated by DRP1 and Fission-1 (FIS1)^40,41^. Our finding that LPS-induced mitochondrial fission is dependent on paxillin and paxillin tyrosine phosphorylation in the ECs is novel. Ectopic expression of paxillin Y31F and Y118F mutants blocked LPS-induced DRP1 Ser 616 phosphorylation and mitochondrial fission. Further, the LPS-induced DRP1 Ser 616 phosphorylation and mitochondrial fission was blocked by PD98059, an inhibitor of MEK that phosphorylates ERK1/2 (Fig. 6H); thus, providing evidence for ERK1/2 involvement in mitochondrial fission elicited by LPS. Interestingly, LPS stimulated DRP1 Ser 616 phosphorylation at 30 min that preceded the physiological responses such as mitochondrial fission, mtROS production, VE-cadherin phosphorylation, and permeability changes (Figs. 1, 3, 9). Our co-immunoprecipitation studies show potential association between paxillin, DRP1 and ERK after LPS challenge of HLMVECs and ecto-expression of paxillin Y31 or Y118 mutant reduced the association between paxillin, DRP1 and ERK1. It is unclear if the increased association between paxillin, DRP1 and ERK1/2 is direct or indirect via other proteins, including actin.

In addition to paxillin, cellular cytoskeletal components such as actin and cofilin, an actin-depolymerizing factor, have been implicated in mitochondrial fission^42,43^. Further, translocation of cofilin to mitochondria and interaction with DRP1 was essential for erucin-induced mitochondrial fission and apoptosis in human breast cancer cells^44^. DRP1 phosphorylation at Ser616 or dephosphorylation at Ser 637 regulates its translocation to mitochondria and mitochondria fission^36^. In the current study, LPS stimulated DRP1 Ser616 phosphorylation with no change in the status of DRP1 Ser 637 phosphorylation; however, dephosphorylation of DRP1Ser 637 in response to erucin regulated DRP1 translocation to mitochondria^44^. Further, mitochondrial fission and mitophagy are dependent on cofilin-mediated actin depolymerization activity at the mitochondrial fission site^43^. Interestingly, suppression of NADPH oxidase mediated ROS production led to elevated actin polymerization (F-actin) in neutrophils^45^. Thus, it is likely that elevated ROS production could lead to actin depolymerization. However, a plausible link between paxillin-mediated mitoROS in cofilin phosphorylation and actin remodeling is unclear. In addition to DRP1, cofilin is also translocated to mitochondrial outer membrane in breast cancer cells; however, it is unclear if paxillin is translocated to mitochondria in response to LPS.

The two major sources of cellular ROS are NOX family proteins and mitochondria. The NOX2- and NOX4-derived ROS in ECs are known to regulate vascular function; however, excess accumulation of ROS leads to vascular dysfunction^46,47^. In human lung ECs, the effect of HGF- or S1P-induced p47^*phox*^ activation and ROS accumulation on lamellipodia formation is known to be regulated by paxillin tyrosine phosphorylation indicating a role for NOX2^14^. Moreover, interaction between TLR4 and NOX4 was shown to be essential for LPS-induced ROS in ECs and smooth muscle cells^48^. In addition to NOX, several studies have shown that mitochondria and mtROS are immunomodulators^49–51^ and suppression of mtROS alleviated inflammation^52^. However, the role of mtROS in regulating endothelial barrier function is unclear. Here, we have demonstrated for the first time that LPS-induced paxillin tyrosine phosphorylation regulates mtROS production in HLMVECs, and down-regulation of paxillin with siRNA or paxillin function with the Y31 and Y118 mutants reduces LPS-induced mtROS generation. Furthermore, LPS-induced mtROS was dependent on the mitochondrial fission. Here, we have demonstrated that inhibition of mitochondrial fission with a DRP1 specific inhibitor, P110^38^ markedly suppressed mtROS generation in LPS-stimulated HLMVECs. A similar inhibition of LPS-induced mtROS generation by Mdivi-1 and *DRP1* knockdown attenuating the production of pro-inflammatory mediators was reported in microglial cells^53^. Additionally, LPS-induced mitochondrial fission and mtROS production was attenuated by PD98059, an inhibitor of MEK suggesting a role for ERK1/2. While this study shows mitochondrial fission as an upstream regulator of mtROS generation, in certain cell types such as primary hippocampal neurons mtROS seems to regulate beta-amyloid (Aβ) induced mitochondrial morphological changes. Aβ-mediated mitochondrial granular shape in hippocampal neurons was dependent on mtROS as mitoTEMPO restored tubular mitochondrial morphology suggesting a reciprocal relationship between mtROS and mitochondrial dynamics in neurodegeneration^38^. The present study does not address which of the pathways in the mtROS production are modulated by paxillin and paxillin tyrosine phosphorylation. In the mitochondria, escape of electrons from the mitochondrial electron transport chain (ETC) results in the formation of superoxide (O_2_^.-^)^54^, while NOX4 localized in the mitochondria generates hydrogen peroxide (H_2_O_2_)^55,56^. While we were able to demonstrate LPS-induced mtROS between 6 and 24 h post-LPS challenge, there was no increase of ROS in the cytoplasm and nuclear compartments as determined by targeted pHyPer-cytosolic and pHyPer-nuclear ROS sensors (Fig. 1). It is conceivable that LPS stimulated ROS production in the cytoplasmic compartment earlier to 6 h, and we did not determine ROS generation induced by LPS between 1 and 5 h, and therefore cannot rule out ROS production in the cytoplasm. Further studies are necessary to address the precise role of paxillin and paxillin tyrosine phosphorylation in ETC and/or NOX4 dependent mtROS production in response to a pro-inflammatory stimulus in the endothelium.

Maintenance of endothelial barrier integrity is crucial for vessel wall homeostasis and lung function under normal and pathological conditions such as sepsis, ARDS, bacterial lung infection and ventilator-induced lung injury. Increased ROS production mediated by NOX2/NOX4 has been linked to endothelial barrier disruption and pulmonary edema, a hallmark of ARDS or sepsis-induced lung inflammatory injury^46^. Earlier studies have suggested that mtROS regulate VEGFR transactivation and down-stream signaling^57^, endothelial sprouting^58,59^, TNF-α-mediated apoptosis^60^, and endothelial inflammation^61^. However, the role of mtROS in endothelial barrier dysfunction in lung inflammatory injury is unclear. Our study suggests a causal relationship between LPS-induced mitochondrial fission, mtROS and endothelial permeability. The LPS-induced endothelial barrier dysfunction, as determined by TER and FITC-dextran techniques, was blunted by paxillin mutants, DRP1 inhibition with P110 and scavenging mtROS with mitoTEMPO. One potential mechanism of LPS-induced endothelial hyper-permeability is by disassembly of adherens junction proteins including claudin, occludin, and VE-cadherin^62^. LPS and other inflammatory mediators stimulate VE-cadherin phosphorylation at Y658, Y685 and Y731 that induces vascular permeability and leukocyte extravasation. Inhibition of DRP1 and scavenging mtROS attenuated LPS-induced VE-cadherin Y658 phosphorylation and endothelial permeability. These results demonstrate for the first time a causal link between mitochondrial fission, and mtROS in modulating VE-cadherin phosphorylation at the adherens junction (AJ) by LPS and modulating endothelial permeability. In addition to endothelial permeability, mtROS has been shown to regulate pro-inflammatory responses of macrophages to bacterial infection through disulfide linkage of nuclear factor kB (NF-kB) essential modulator (NEMO)^63^, expression of pro-inflammatory mediators in microglial cells^64^, and VEGF-induced endothelial migration through Rac1^65^. Further, a recent study suggests a role for NOX-derived ROS in the cytosol in the generation of mtROS in coronary endothelium^59^; however, in our study, LPS challenge between 6–24 h stimulated predominantly mtROS and not the cytosolic or nuclear ROS in HLMVECs, which was regulated by paxillin tyrosine phosphorylation dependent DRP1 activation. While the mtROS dependent signaling pathway(s) involved in VE-cadherin Y658 phosphorylation/dephosphorylation is yet to be defined, several tyrosine phosphatases including VE-PTP^66^, SHP2^67^, and PTPN14^68^ have been implicated in dephosphorylation of VE-cadherin at AJs to maintain a non-leaky endothelial barrier. It has been shown that ROS inhibits phosphatases especially protein tyrosine phosphatases^69^ and it is possible that mtROS generated by LPS inhibits VE-PTP, SHP2 or PTPN14, and maintains VE-cadherin tyrosine phosphorylation that results in delayed recovery of AJs and barrier integrity. Further studies are necessary to determine mechanism(s) involved in mtROS mediated regulation of VE-cadherin phosphorylation/dephosphorylation status in the AJs of endothelial cells exposed to inflammatory mediators such as LPS or thrombin.

**Figure 10:**
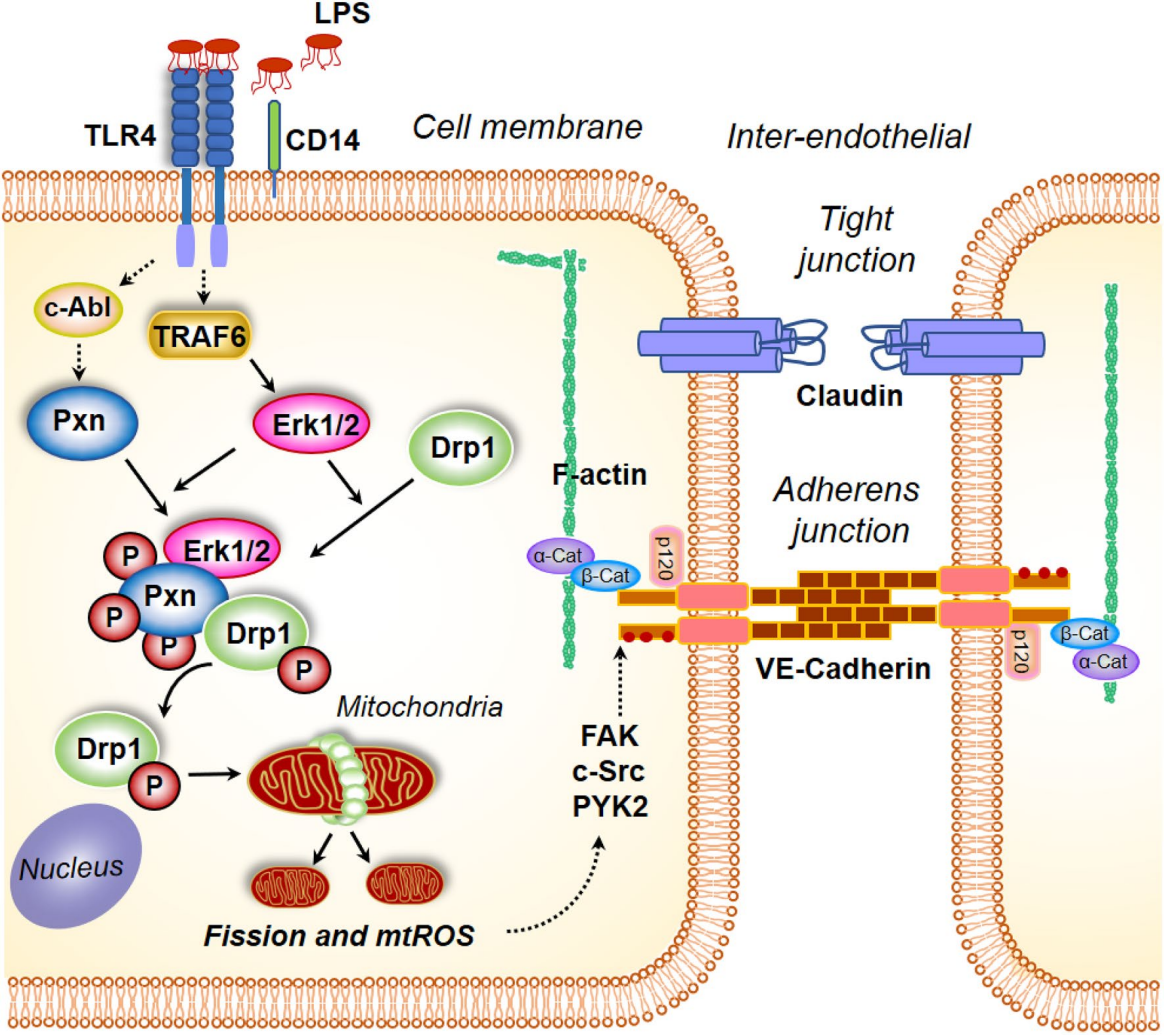
Model of paxillin and DRP1 activation by LPS in mitochondrial fission, mitochondrial ROS generation and endothelial permeability. Stimulation of endothelial cells with lipopolysaccharide (LPS) results in changes in paxillin phosphorylation, which is essential for DRP1 phosphorylation by ERK. Activation of DRP1 results in its translocation to mitochondria resulting in mitochondrial fission accompanied by enhanced mitochondrial ROS. Blocking paxillin or DRP1 phosphorylation attenuates mitochondrial ROS generation and VE-cadherin phosphorylation status. Similarly, blocking mitochondrial ROS attenuated LPS-induced VE-cadherin phosphorylation and endothelial permeability.

In conclusion, in this study we have defined a novel pathway of paxillin tyrosine phosphorylation mediated mitochondrial fission, which regulates LPS-mediated mtROS production and endothelial barrier dysfunction (Fig. 10). Further, we have demonstrated that the mtROS-mediated endothelial barrier dysfunction is due to aberrant dephosphorylation of VE-cadherin at AJs. Thus, our results suggest that regulating mitochondrial dynamics and/or mtROS may be a novel therapeutic approach to ameliorate pulmonary leak in inflammatory lung pathologies such as ARDS, sepsis and ventilator-induced lung injury.

## Materials and methods

### Materials

Human lung microvascular endothelial cells (HLMVECs) (catalog: cc-2583) and endothelial basal media (EBM-2) (catalog: 3162) were obtained from Lonza (San Diego, CA). FuGENE HD (catalog: E2311) transfection reagent was from Promega Corporation (Madison, WI). PD98059 (catalog: P215-1MG) was purchased from Sigma-Aldrich (Saint Louis, MO). Bovine serum albumin (catalog: sc-2323) was obtained from Santa Cruz Biotechnology (Dallas, TX). Thirty-five mm poly-D-lysine-coated glass-bottomed dishes were from MatTek (Ashland, MA, USA). MitoSOX and MitoTracker Red were from Fisher Scientific (Waltham, MA). Paxillin, Y31, Y118 phosphor-paxillin, Erk1/2, phosphor-Erk, DRP1 and S616 phospho-DRP1 antibodies were from Cell Signaling Technology (Danvers, MA).

### Endothelial cell culture

HLMVECs cultured in complete media (EBM-2) containing 10% fetal bovine serum (FBS), growth factors (provided as a kit from Lonza (San Diego, CA) and 1% Penicillin/Streptomycin were maintained at 37 °C and 5% CO2 and grown to contact-inhibited monolayers that revealed typical cobblestone morphology. Cells were then detached with 0.05% trypsin and resuspended in fresh complete EBM-2 medium, and cultured on gold electrodes for electrical resistance determinations (transendothelial electrical resistance TER), or glass coverslips for fluorescent microscopy studies, or on 35, 60 or 100-mm culture dishes for preparation of cell lysates and Western blot analysis.

### Measurement of transendothelial electrical resistance

Transendothelial electrical resistance (TER) was measured in an electrical cell-substrate impedance sensing system (Applied Biophysics, Troy, NY) as described previously^14^. Briefly, HLMVECs were grown to ∼ 95% confluence in polycarbonate wells containing gold electrodes connected to a phase-sensitive lock-in amplifier. Electrodes containing cells were placed in an electrical cell-substrate impedance incubator for 1 h to stabilize basal electrical resistance. The total electrical resistance across the endothelial monolayer was determined by the combined resistance between the basal surface of the cell and the electrode, providing a measure of alterations in cell–cell or cell–matrix adhesion. In the experiments assessing the time course of the response, TER is expressed as normalized resistance (i.e., ratio of resistance at a given time to resistance at “zero” time).

### Plasmids and transfection

Enhanced green fluorescent protein (EGFP)-tagged wild type paxillin and Y31F, Y118F mutants were prepared as described previously^13^. ROS biosensors targeted to cytosol, mitochondria, and nucleus were obtained from Evrogen (Moscow, Russia). Constructs were added to HLMVECs grown to ∼ 80% confluence in EBM-2-MV growth media (Lonza) supplemented with 10% FBS. After overnight culture, the medium was replaced with fresh complete medium and cells were cultured for additional 48 h before experimentation.

### Live cell ROS imaging

Live cell ROS detection was performed with a Carl Zeiss 780 Confocal microscopy with cells either transfected with different ROS biosensors localized at cytosol, mitochondria, or nucleus, or cells stained with mitochondrial ROS detector, MitoSOX. Forty eight hours after transfection, HLMVECs cultured in MatTek (Ashland, MA, USA) 35 mm poly-D-lysine-coated glass-bottomed dish were changed with fresh medium containing 2% FBS, and the cell culture dish was mounted onto con-focal microscopy equipped with a temperature-controlled chamber supplied with humidified 5% CO2. Thirty minutes after the cells adapted to the chamber environment, basal ROS images were acquired with 63 × /1.40 oil objective at excitation wavelength of 488 nm or 587 nm for detection of ROS biosensor or MitoSOX signal, respectively. After basal ROS acquired, LPS was added into cell culture medium, followed by image acquisition at different time points. Quantification of ROS was performed as previously described.

### Live cell mitochondria visualization and mitochondrial morphology quantification

Mitochondria visualization was carried out by live cells labelled with MitoTracker Red. This was achieved by incubating the cells with culture media containing 20 nM dye for 30 min within the culture incubator, followed by washing 3 times with pre-warmed phosphate-buffered saline. Images were obtained with Carl Zeiss 780 Confocal microscopy as described above with excitation wavelength of 587 nm and 590–660 emission filter set. Analysis of image was carried out with ImageJ software. Analysis of mitochondrial network morphology was preceded with image pre-processing steps including unsharp mask, enhancement of local contrast, binary making and skeletonization. These processes were integrated into an Image J Macro file and were applied for all images processing. All pixels within a skeleton are then grouped into three categories: end point pixels, slab pixels, and junction pixels. Pixels spatial relationships were used to measure the length of each branch and the number of branches in each skeletonized feature. To simplify the analysis, numbers of individual structures (rod, punctate, large/round), networks and branches, and length of rod and branch, were used for quantification to define mitochondrial morphology.

### Immunoblotting and immunoprecipitation

Immunoblotting and immunoprecipitation (IP) studies were performed as described previously^14,70^. In brief, after appropriate treatments, cells were pelleted in ice-cold PBS, lysed in standard lysis buffer (Cell Signaling, Beverly, MA), and sonicated. Lysates were then centrifuged at 1,000×g for 10 min at 4 °C, supernatants were collected, and protein assayed using BCA protein assay kit. For IP experiments, equal amounts of protein (1 mg) from each sample were pre-cleared with control IgG conjugated to Protein A/G agarose beads at 4 °C for 1 h, supernatants were collected and incubated overnight with primary antibody coupled with Protein A/G agarose beads at 4 °C. Next day, the samples were centrifuged at 1,000×g for 1 min in a microfuge centrifuge and the pellet containing the agarose beads were washed three times with lysis buffer at room temperature. After centrifugation at 1,000×g for 1 min, the beads were collected by removing supernatant buffer, and 40 µl of SDS sample buffer (100 mM Tris–HCl pH 6.8, 4% SDS, 0.1% bromophenol blue, 20% glycerol, 200 mM DTT] were added to the beads and boiled. Lysates were then subjected to 10% SDS-PAGE followed by Western blotting. Proteins were detected by immunoblotting using appropriate primary antibodies, and HRP-conjugated anti-rabbit or anti-mouse secondary antibodies. Band intensities were quantified by densitometry using Image J software.

### Statistical analysis

Results are expressed as means ± SD of 3–5 independent experiments. Statistical significance was assessed by ANOVA followed by multiple comparisons with Bonferroni corrections. Statistical significance was defined at p < 0.05.

## Supplementary Information

Supplementary Information.

## Abbreviations

AJ: Adherens junction
ARDS: Acute respiratory distress syndrome
BCA: Bicinchoninic acid
DTT: Dithiothreitol
ETC: Electron transport chain
FAK: Focal adhesion kinase
HGF: Hepatocyte growth factor
HRP: Horseradish peroxidase
IgG: Immunoglobulin gamma
IP: Immunoprecipitation
LD: Leucine-rich domain
LIM: Zinc-binding domain present in Lin-11, Isl-1, Mec-3
LPS: Lipopolysaccharide
MtROS: Mitochondrial ROS
NOX: Nicotinamide adenine dinucleotide phosphate oxidase
PBS: Phosphate buffered saline
ROS: Reactive oxygen species
SDS: Sodium dodecyl sulphate
TER: Transendothelial electrical resistance
PAGE: Polyacrylamide gel electrophoresis
SH3: SRC homology 3
TLR2: Toll-like receptor 2
TLR4: Toll-like receptor 4
VEGFR: Vascular endothelial growth factor receptor

## References

[1] Brown MC, Turner CE (2004). Paxillin: adapting to change. Physiol. Rev..

[2] Turner CE (2000). Paxillin interactions. J. Cell Sci..

[3] Iwasaki T (2002). Involvement of phosphorylation of Tyr-31 and Tyr-118 of paxillin in MM1 cancer cell migration. Int. J. Cancer.

[4] Petit V (2000). Phosphorylation of tyrosine residues 31 and 118 on paxillin regulates cell migration through an association with CRK in NBT-II cells. J. Cell Biol..

[5] Salgia R (1999). Expression of the focal adhesion protein paxillin in lung cancer and its relation to cell motility. Oncogene.

[6] Turner CE (1999). Paxillin LD4 motif binds PAK and PIX through a novel 95-kD ankyrin repeat, ARF-GAP protein: A role in cytoskeletal remodeling. J. Cell Biol..

[7] Teranishi S, Kimura K, Nishida T (2009). Role of formation of an ERK-FAK-paxillin complex in migration of human corneal epithelial cells during wound closure in vitro. Invest. Ophthalmol. Vis. Sci..

[8] Schaller MD (2001). Paxillin: A focal adhesion-associated adaptor protein. Oncogene.

[9] Webb DJ (2004). FAK-Src signalling through paxillin, ERK and MLCK regulates adhesion disassembly. Nat. Cell Biol..

[10] Yano H (2000). Paxillin alpha and Crk-associated substrate exert opposing effects on cell migration and contact inhibition of growth through tyrosine phosphorylation. Proc. Natl. Acad. Sci. USA.

[11] Brown MC, Cary LA, Jamieson JS, Cooper JA, Turner CE (2005). Src and FAK kinases cooperate to phosphorylate paxillin kinase linker, stimulate its focal adhesion localization, and regulate cell spreading and protrusiveness. Mol. Biol. Cell.

[12] Roy S, Ruest PJ, Hanks SK (2002). FAK regulates tyrosine phosphorylation of CAS, paxillin, and PYK2 in cells expressing v-Src, but is not a critical determinant of v-Src transformation. J. Cell Biochem..

[13] Fu P (2015). c-Abl mediated tyrosine phosphorylation of paxillin regulates LPS-induced endothelial dysfunction and lung injury. Am. J. Physiol. Lung Cell Mol. Physiol..

[14] Fu P (2015). Role played by paxillin and paxillin tyrosine phosphorylation in hepatocyte growth factor/sphingosine-1-phosphate-mediated reactive oxygen species generation, lamellipodia formation, and endothelial barrier function. Pulm Circ..

[15] Choy KW, Lau YS, Murugan D, Vanhoutte PM, Mustafa MR (2018). Paeonol attenuates LPS-induced endothelial dysfunction and apoptosis by inhibiting BMP4 and TLR4 signaling simultaneously but independently. J. Pharmacol. Exp. Ther..

[16] Pawar RD (2009). Bacterial lipopeptide triggers massive albuminuria in murine lupus nephritis by activating Toll-like receptor 2 at the glomerular filtration barrier. Immunology.

[17] Kratzer E (2012). Oxidative stress contributes to lung injury and barrier dysfunction via microtubule destabilization. Am. J. Respir. Cell Mol. Biol..

[18] Gandhirajan RK (2013). Blockade of NOX2 and STIM1 signaling limits lipopolysaccharide-induced vascular inflammation. J. Clin. Invest..

[19] Fu P (2009). Amifostine reduces lung vascular permeability via suppression of inflammatory signalling. Eur. Respir. J..

[20] Qi D (2017). Vaspin protects against LPSinduced ARDS by inhibiting inflammation, apoptosis and reactive oxygen species generation in pulmonary endothelial cells via the Akt/GSK3beta pathway. Int. J. Mol. Med..

[21] Vieira LD (1864). Oxidative stress induced by prenatal LPS leads to endothelial dysfunction and renal haemodynamic changes through angiotensin II/NADPH oxidase pathway: Prevention by early treatment with alpha-tocopherol. Biochim. Biophys. Acta Mol. Basis Dis..

[22] Han JE, Choi JW (2012). Control of JNK for an activation of NADPH oxidase in LPS-stimulated BV2 microglia. Arch. Pharm. Res..

[23] Check J (2010). Src kinase participates in LPS-induced activation of NADPH oxidase. Mol. Immunol..

[24] Singel KL, Segal BH (2016). NOX2-dependent regulation of inflammation. Clin. Sci. (Lond).

[25] Rada B, Leto TL (2008). Oxidative innate immune defenses by Nox/Duox family NADPH oxidases. Contrib. Microbiol..

[26] Leto TL, Geiszt M (2006). Role of Nox family NADPH oxidases in host defense. Antioxid. Redox. Signal.

[27] Yang CS (2009). NADPH oxidase 2 interaction with TLR2 is required for efficient innate immune responses to mycobacteria via cathelicidin expression. J. Immunol..

[28] Li Q, Li J, Liu Y, Zhang M, Chen C (2018). Anagliptin prevents apoptosis of human umbilical vein endothelial cells by modulating NOX-4 signaling pathways. Biomed. Pharmacother..

[29] Lim SG, Suk K, Lee WH (2020). LETMD1 regulates phagocytosis and inflammatory responses to lipopolysaccharide via reactive oxygen species generation and NF-kappaB activation in macrophages. J. Immunol..

[30] Reddy SS (2016). Coagulin-L ameliorates TLR4 induced oxidative damage and immune response by regulating mitochondria and NOX-derived ROS. Toxicol. Appl. Pharmacol..

[31] Zhou K (2019). Schaftoside ameliorates oxygen glucose deprivation-induced inflammation associated with the TLR4/Myd88/Drp1-related mitochondrial fission in BV2 microglia cells. J. Pharmacol. Sci..

[32] Shi J (2018). Phosphatidylinositol 3-kinase-mediated HO-1/CO represses Fis1 levels and alleviates lipopolysaccharide-induced oxidative injury in alveolar macrophages. Exp. Ther. Med..

[33] Baker B, Maitra U, Geng S, Li L (2014). Molecular and cellular mechanisms responsible for cellular stress and low-grade inflammation induced by a super-low dose of endotoxin. J. Biol. Chem..

[34] Perez-Pinzon MA, Stetler RA, Fiskum G (2012). Novel mitochondrial targets for neuroprotection. J. Cereb. Blood Flow Metab..

[35] Chang CR, Blackstone C (2007). Cyclic AMP-dependent protein kinase phosphorylation of Drp1 regulates its GTPase activity and mitochondrial morphology. J. Biol. Chem..

[36] Cribbs JT, Strack S (2007). Reversible phosphorylation of Drp1 by cyclic AMP-dependent protein kinase and calcineurin regulates mitochondrial fission and cell death. EMBO Rep..

[37] Cereghetti GM (2008). Dephosphorylation by calcineurin regulates translocation of Drp1 to mitochondria. Proc. Natl. Acad. Sci. USA.

[38] Qi X, Qvit N, Su YC, Mochly-Rosen D (2013). A novel Drp1 inhibitor diminishes aberrant mitochondrial fission and neurotoxicity. J. Cell Sci..

[39] Kashatus JA (2015). Erk2 phosphorylation of Drp1 promotes mitochondrial fission and MAPK-driven tumor growth. Mol. Cell.

[40] Atkins K, Dasgupta A, Chen KH, Mewburn J, Archer SL (2016). The role of Drp1 adaptor proteins MiD49 and MiD51 in mitochondrial fission: Implications for human disease. Clin. Sci. (Lond).

[41] Pernas L, Scorrano L (2016). Mito-morphosis: mitochondrial fusion, fission, and cristae remodeling as key mediators of cellular function. Annu. Rev. Physiol..

[42] Hatch AL, Gurel PS, Higgs HN (2014). Novel roles for actin in mitochondrial fission. J. Cell Sci..

[43] Li GB (2018). Mitochondrial fission and mitophagy depend on cofilin-mediated actin depolymerization activity at the mitochondrial fission site. Oncogene.

[44] Li G (2015). Mitochondrial translocation and interaction of cofilin and Drp1 are required for erucin-induced mitochondrial fission and apoptosis. Oncotarget.

[45] Sakai J (2012). Reactive oxygen species-induced actin glutathionylation controls actin dynamics in neutrophils. Immunity.

[46] Fu P (2012). Role of nicotinamide adenine dinucleotide phosphate-reduced oxidase proteins in *Pseudomonas aeruginosa*-induced lung inflammation and permeability. Am. J. Respir. Cell Mol. Biol..

[47] Park HS (2004). Cutting edge: direct interaction of TLR4 with NAD(P)H oxidase 4 isozyme is essential for lipopolysaccharide-induced production of reactive oxygen species and activation of NF-kappa B. J. Immunol..

[48] Park HS, Chun JN, Jung HY, Choi C, Bae YS (2006). Role of NADPH oxidase 4 in lipopolysaccharide-induced proinflammatory responses by human aortic endothelial cells. Cardiovasc. Res..

[49] Weinberg SE, Sena LA, Chandel NS (2015). Mitochondria in the regulation of innate and adaptive immunity. Immunity.

[50] Shekhova E (2020). Mitochondrial reactive oxygen species as major effectors of antimicrobial immunity. PLoS Pathog..

[51] West AP, Shadel GS, Ghosh S (2011). Mitochondria in innate immune responses. Nat. Rev. Immunol..

[52] Kasahara E (2011). Mitochondrial density contributes to the immune response of macrophages to lipopolysaccharide via the MAPK pathway. FEBS Lett..

[53] Haddad JJ, Land SC (2002). Redox/ROS regulation of lipopolysaccharide-induced mitogen-activated protein kinase (MAPK) activation and MAPK-mediated TNF-alpha biosynthesis. Br. J. Pharmacol..

[54] Nickel A, Kohlhaas M, Maack C (2014). Mitochondrial reactive oxygen species production and elimination. J. Mol. Cell Cardiol..

[55] Hirschhauser C (2015). NOX4 in mitochondria: yeast two-hybrid-based interaction with complex I without relevance for basal reactive oxygen species?. Antioxid. Redox. Signal.

[56] Chen K, Thomas SR, Albano A, Murphy MP, Keaney JF (2004). Mitochondrial function is required for hydrogen peroxide-induced growth factor receptor transactivation and downstream signaling. J. Biol. Chem..

[57] Jung HJ (2013). Mitochondrial UQCRB regulates VEGFR2 signaling in endothelial cells. J. Mol. Med. (Berl).

[58] De Bock K, Georgiadou M, Carmeliet P (2013). Role of endothelial cell metabolism in vessel sprouting. Cell Metab.

[59] Shafique E (2017). Mitochondrial redox plays a critical role in the paradoxical effects of NAPDH oxidase-derived ROS on coronary endothelium. Cardiovasc Res.

[60] Hughes G, Murphy MP, Ledgerwood EC (2005). Mitochondrial reactive oxygen species regulate the temporal activation of nuclear factor kappaB to modulate tumour necrosis factor-induced apoptosis: evidence from mitochondria-targeted antioxidants. Biochem. J..

[61] Forrester SJ (2020). Mitochondrial fission mediates endothelial inflammation. Hypertension.

[62] Chan YH, Harith HH, Israf DA, Tham CL (2019). Differential regulation of LPS-mediated VE-cadherin disruption in human endothelial cells and the underlying signaling pathways: A mini review. Front Cell Dev Biol.

[63] Herb M (2019). Mitochondrial reactive oxygen species enable proinflammatory signaling through disulfide linkage of NEMO. Sci. Signal.

[64] Park J (2013). Mitochondrial dynamics modulate the expression of pro-inflammatory mediators in microglial cells. J. Neurochem..

[65] Wang Y (2011). Regulation of VEGF-induced endothelial cell migration by mitochondrial reactive oxygen species. Am. J. Physiol. Cell Physiol..

[66] Juettner VV (2019). VE-PTP stabilizes VE-cadherin junctions and the endothelial barrier via a phosphatase-independent mechanism. J. Cell Biol..

[67] Zhang J (2019). SHP2 protects endothelial cell barrier through suppressing VE-cadherin internalization regulated by MET-ARF1. FASEB J..

[68] Fu P (2020). Phospholipase D2 restores endothelial barrier function by promoting PTPN14-mediated VE-cadherin dephosphorylation. J. Biol. Chem..

[69] Meng TC, Fukada T, Tonks NK (2002). Reversible oxidation and inactivation of protein tyrosine phosphatases in vivo. Mol. Cell.

[70] Usatyuk PV (2013). Coronin 1B regulates S1P-induced human lung endothelial cell chemotaxis: role of PLD2, protein kinase C and Rac1 signal transduction. PLoS ONE.

