# Supplementary material for: Essential role for paxillin tyrosine phosphorylation in LPS-induced mitochondrial fission, ROS generation and lung endothelial barrier loss
Source: Sci Rep. 2021 Sep 2;11:17546. doi: 10.1038/s41598-021-97006-y (PMC8413352; doi:10.1038/s41598-021-97006-y)
Supplement: Supplementary file 1 — Supplementary Information. [file 41598_2021_97006_MOESM1_ESM.pdf]

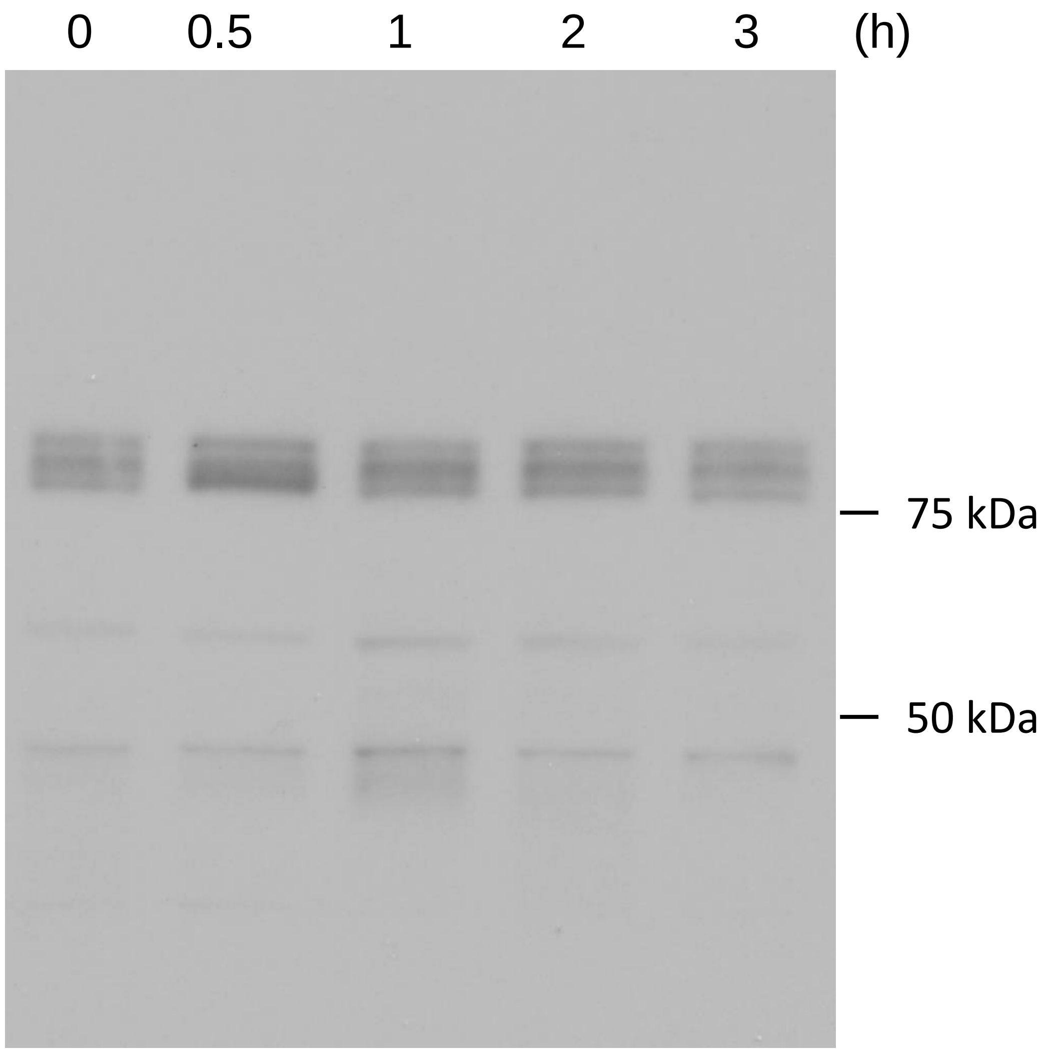

Fig4A p-Drp1 (Ser 616)

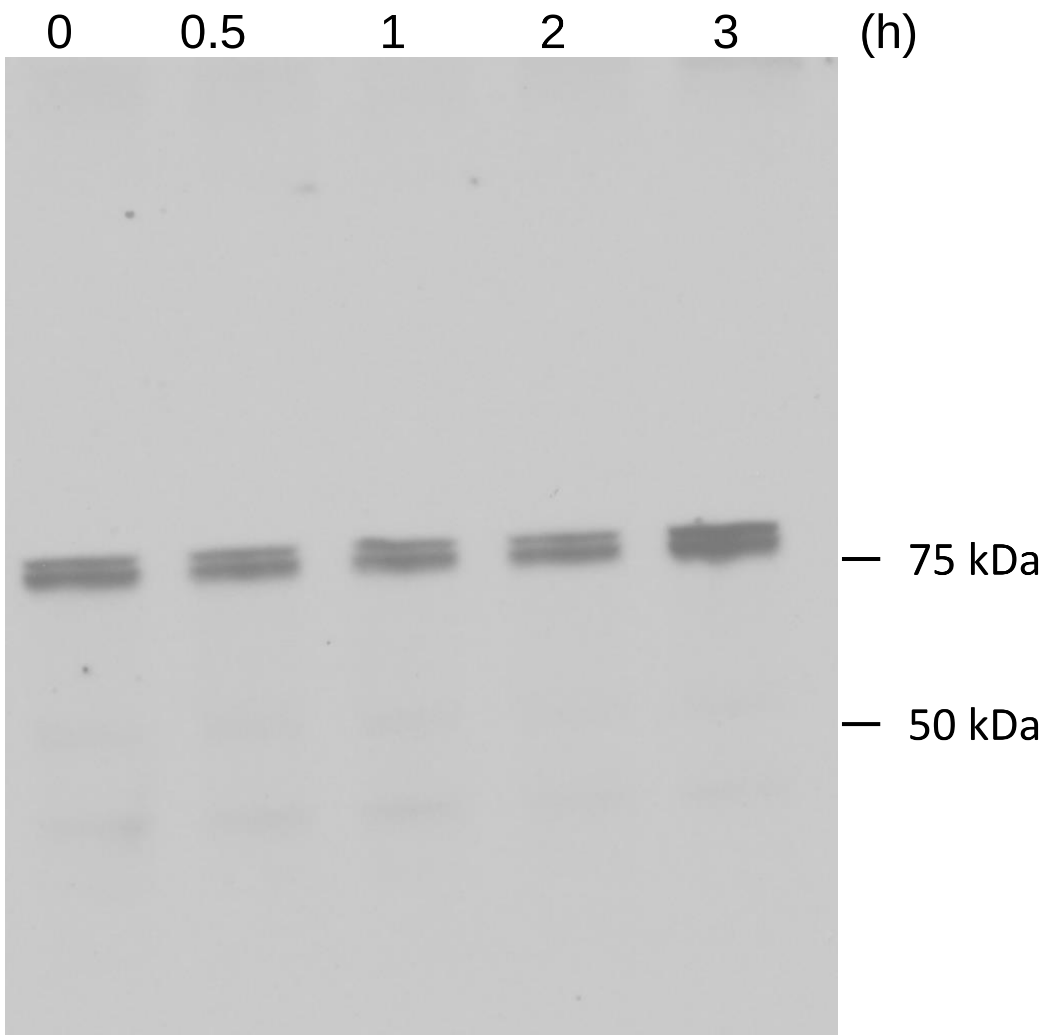

Fig4A Drp1

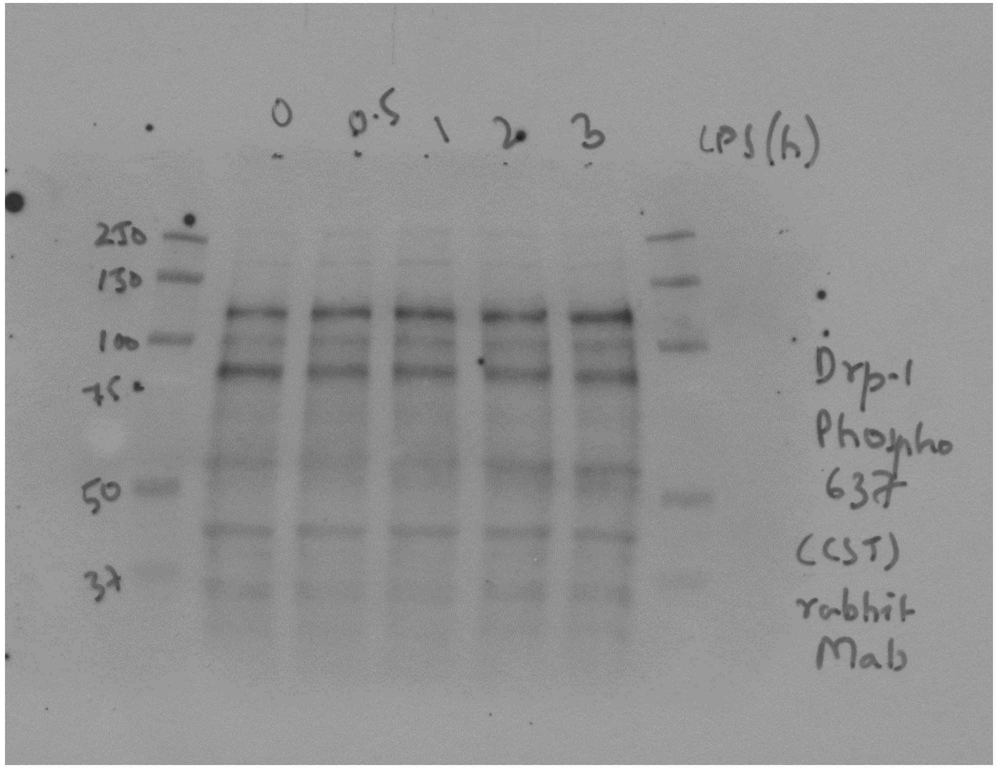

Fig 4A p-Drp1(Ser 637)

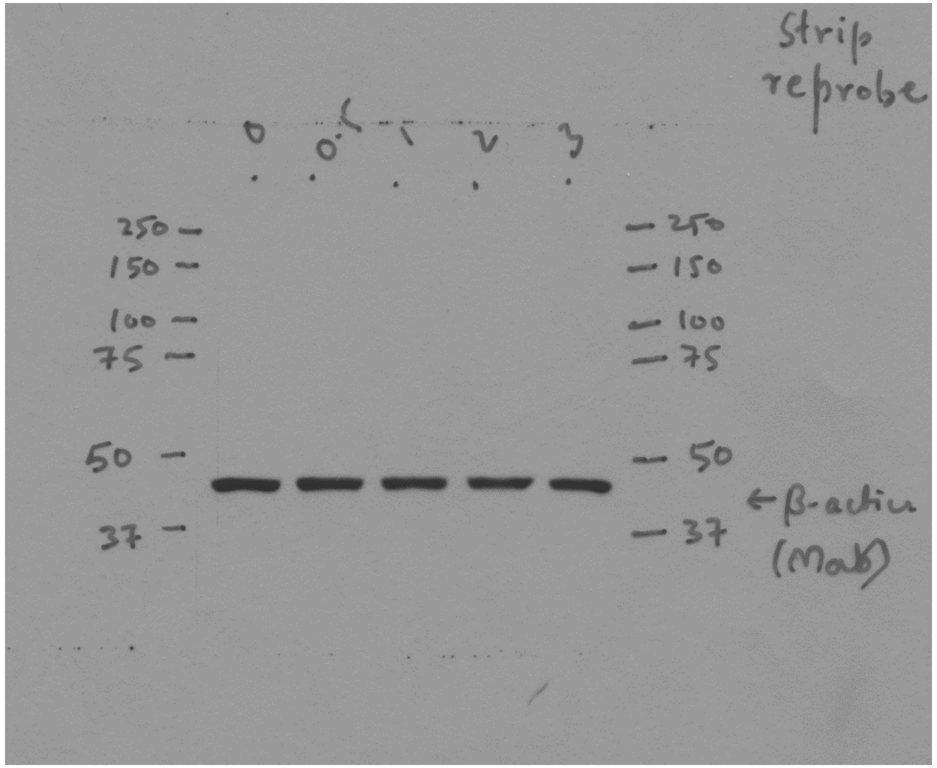

4A actin

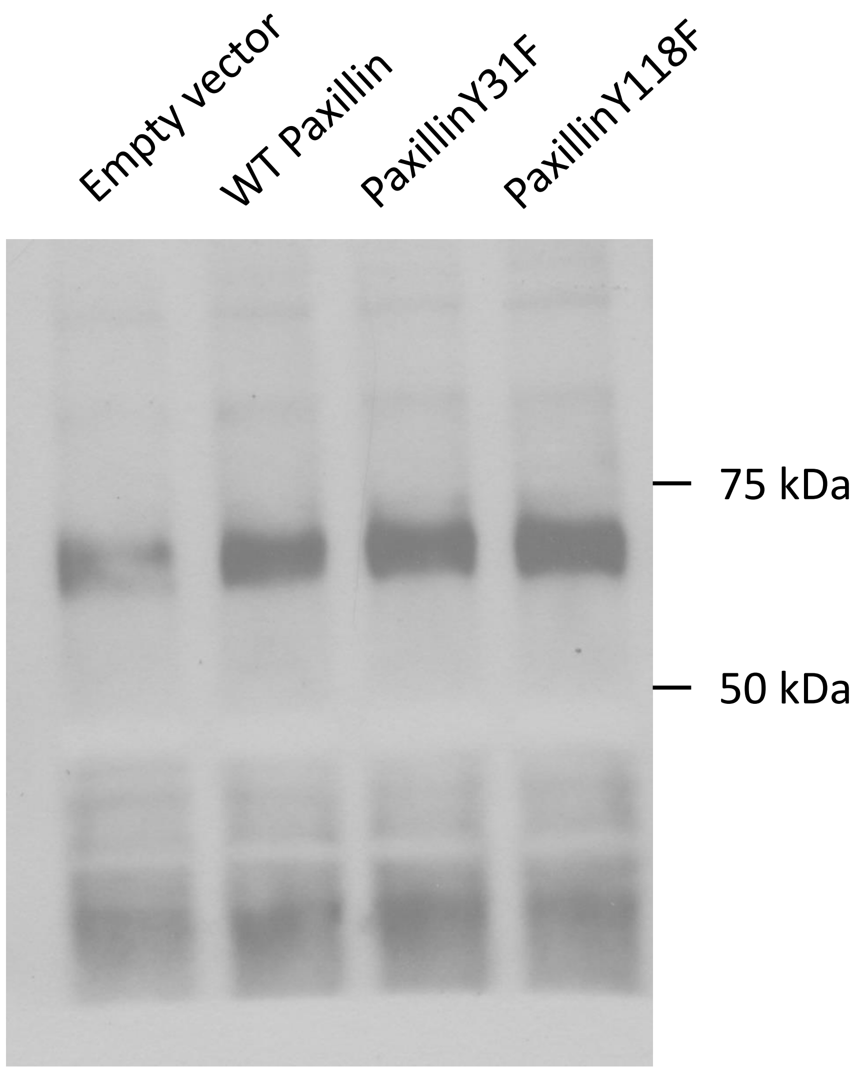

Fig4C Paxillin

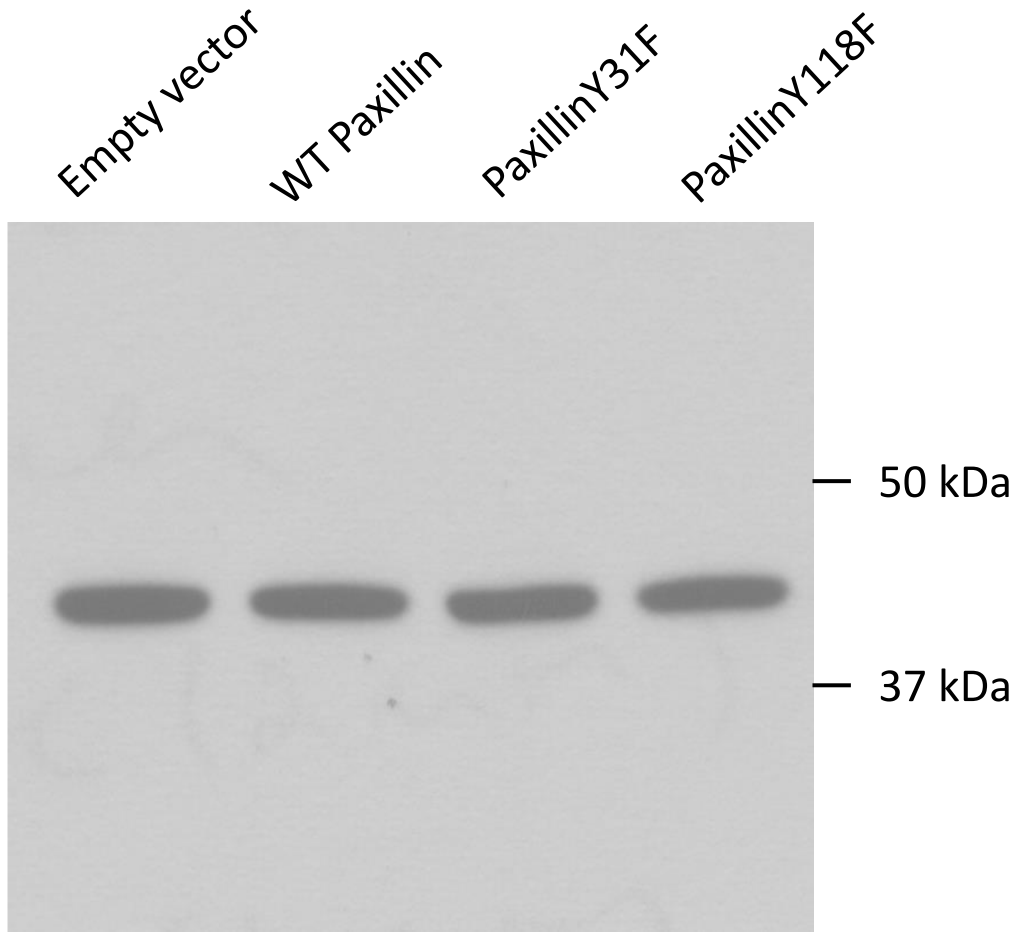

Fig 4C Actin

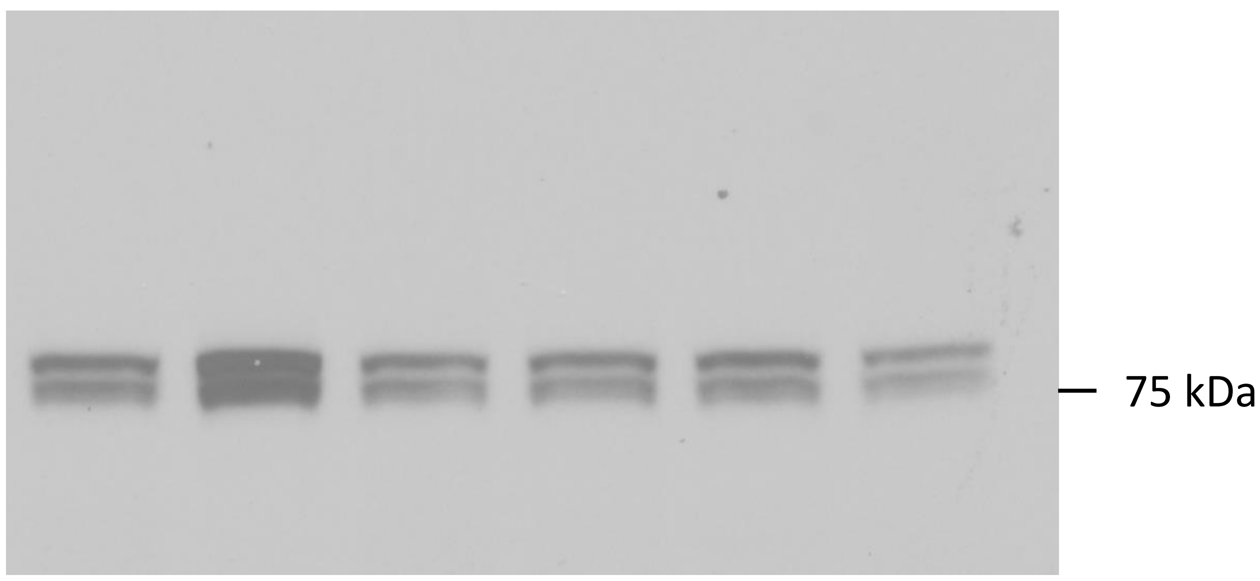

Fig 4E p-Drp1 (Ser616)

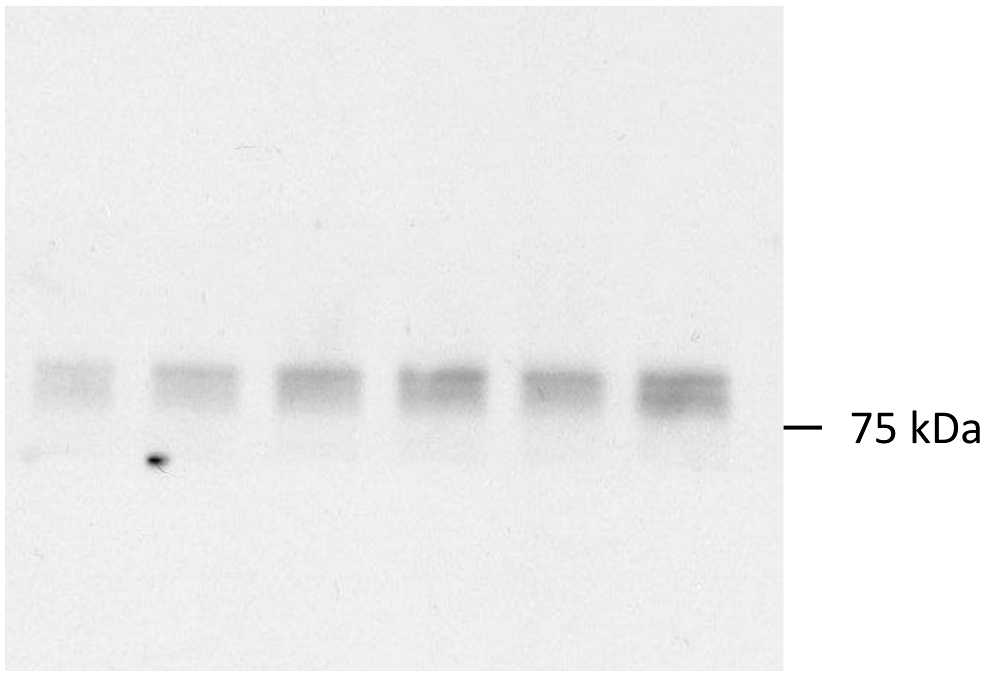

Fig 4E Drp1

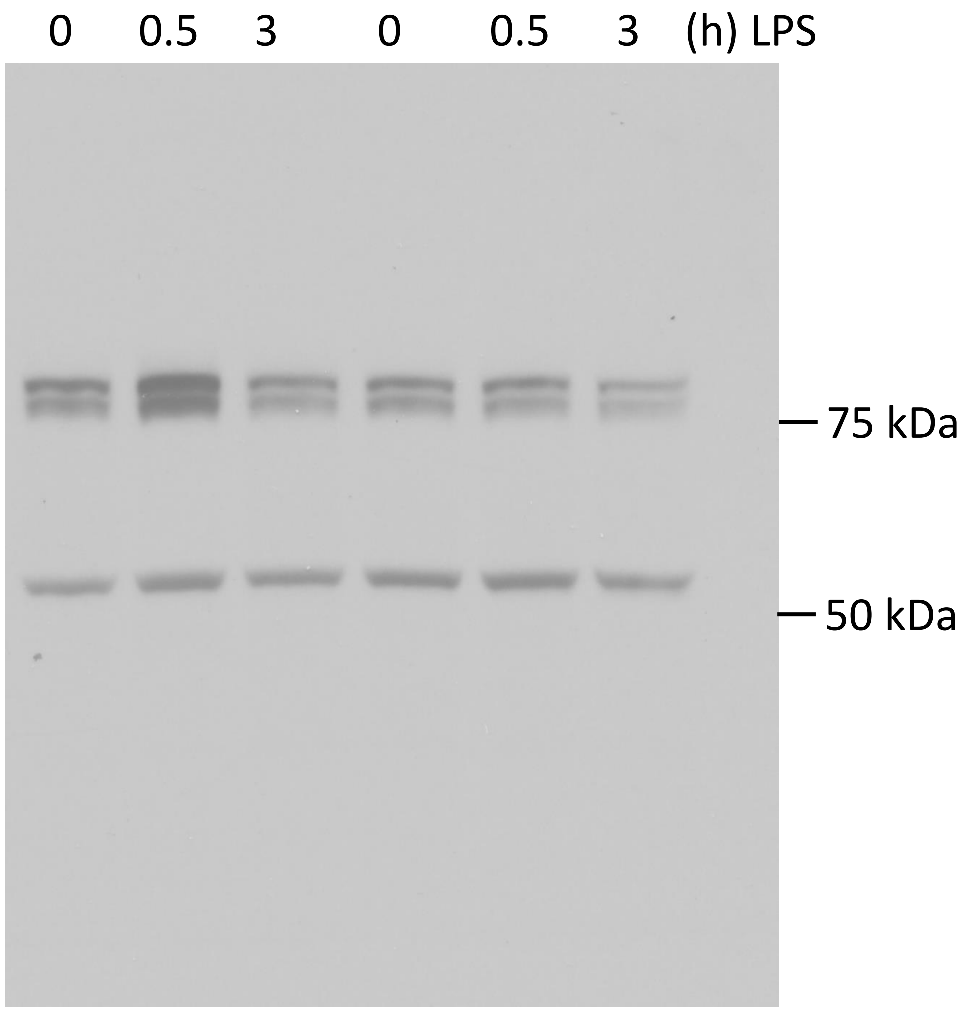

Fig 4G p-Drp1 (Ser616)

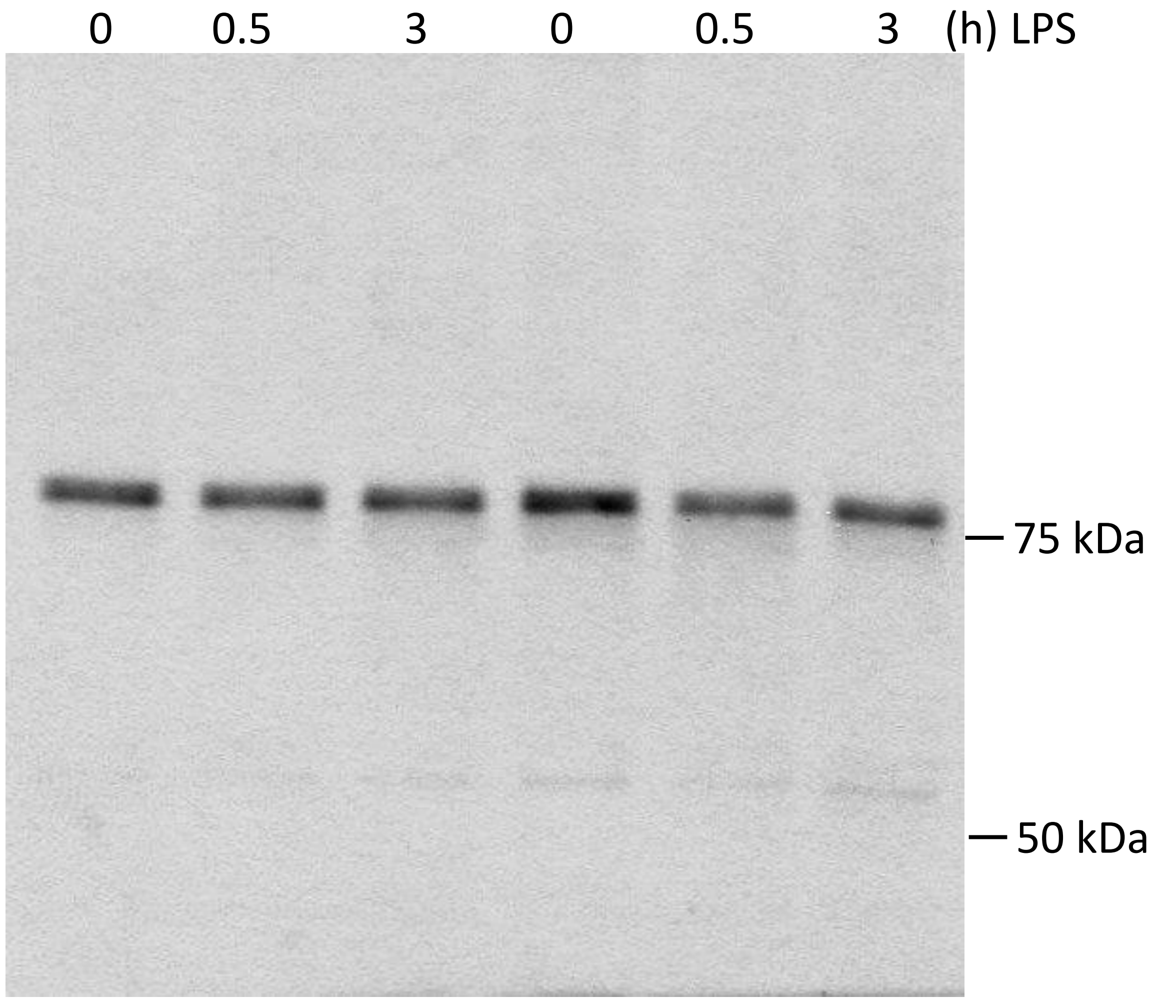

Fig 4G Drp1

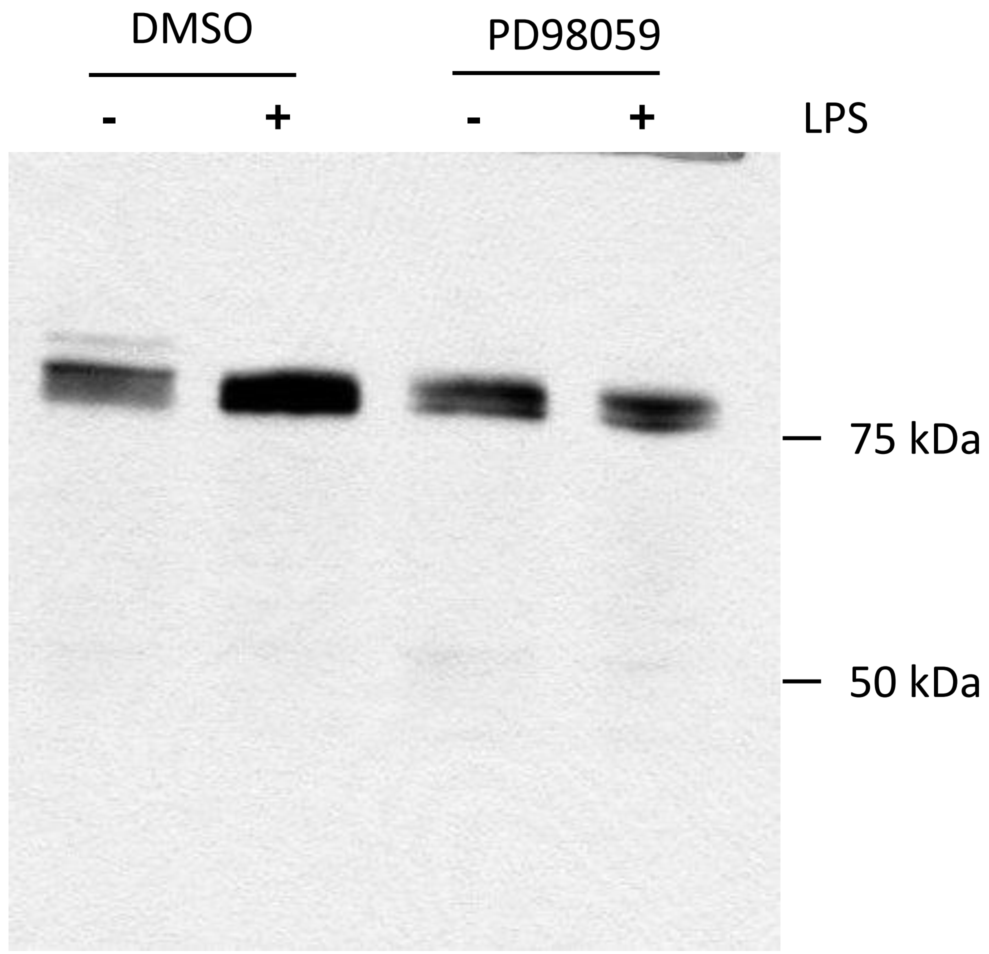

Fig 6A p-Drp1 (Ser616)

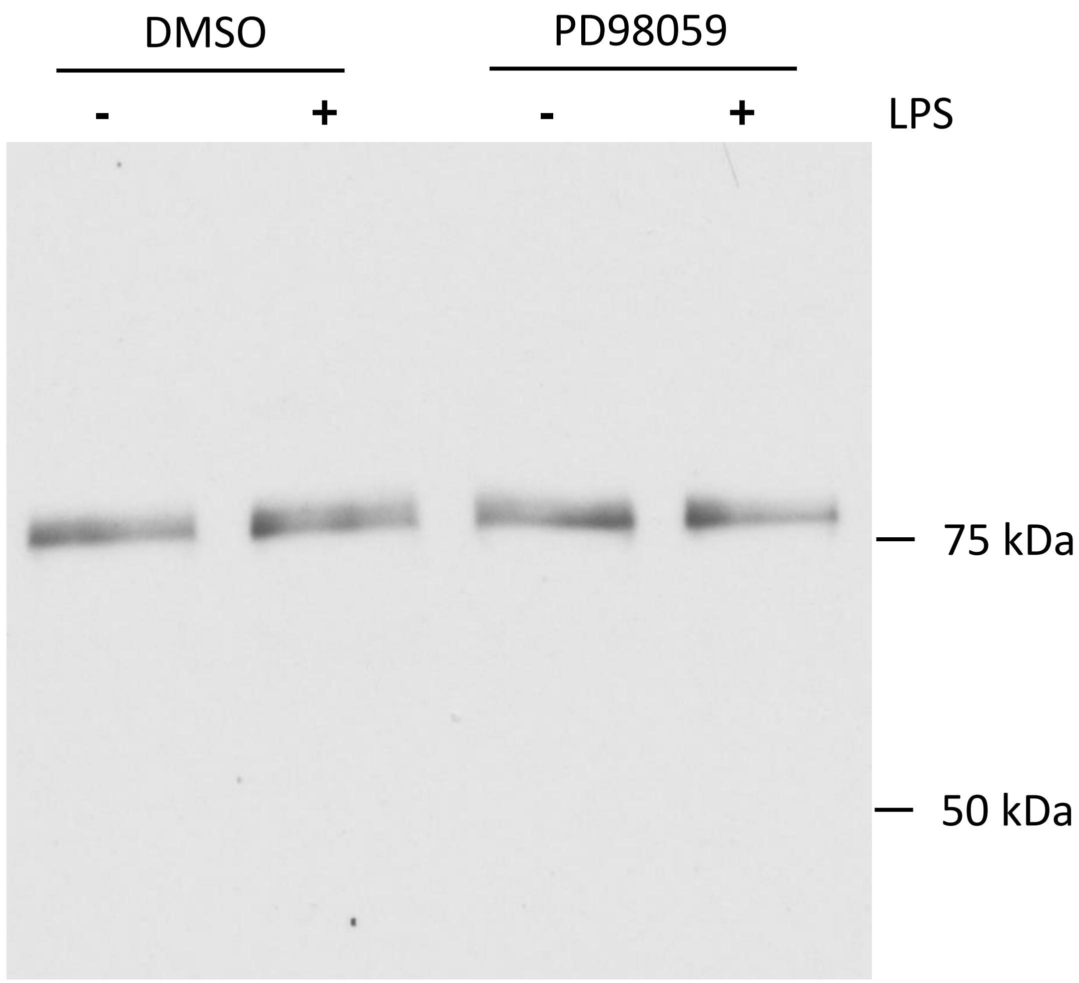

Fig 6A Drp1

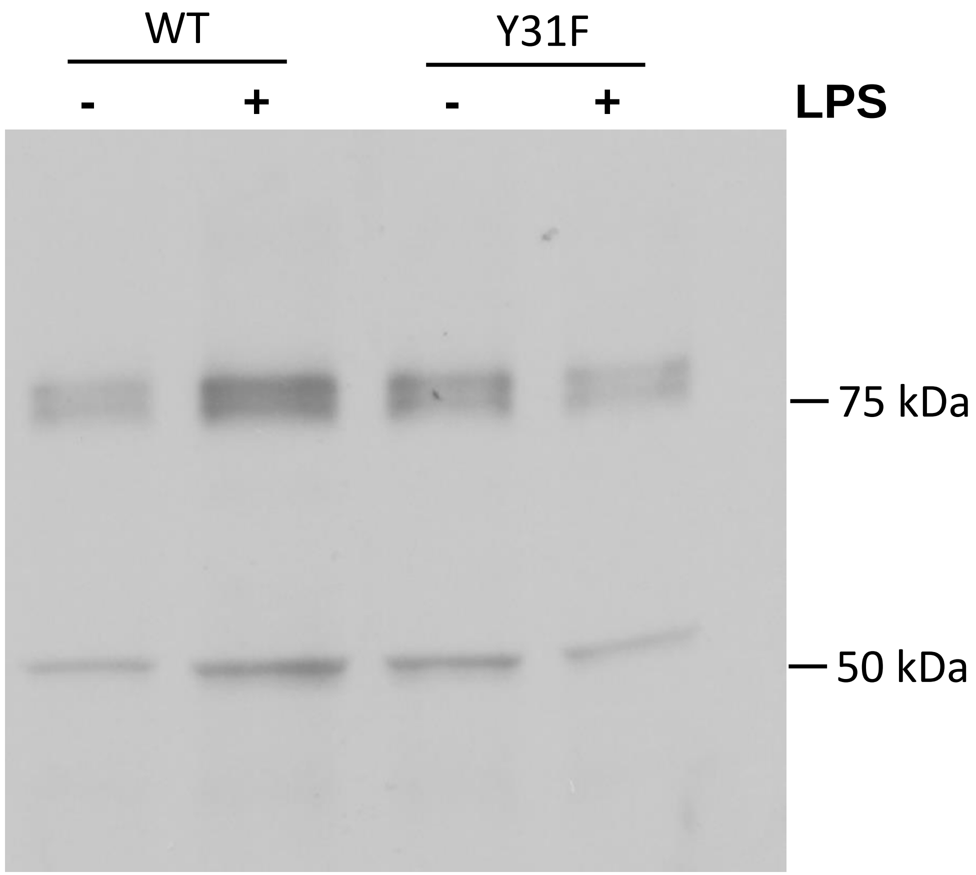

Fig 7A Drp1 (Ser616)

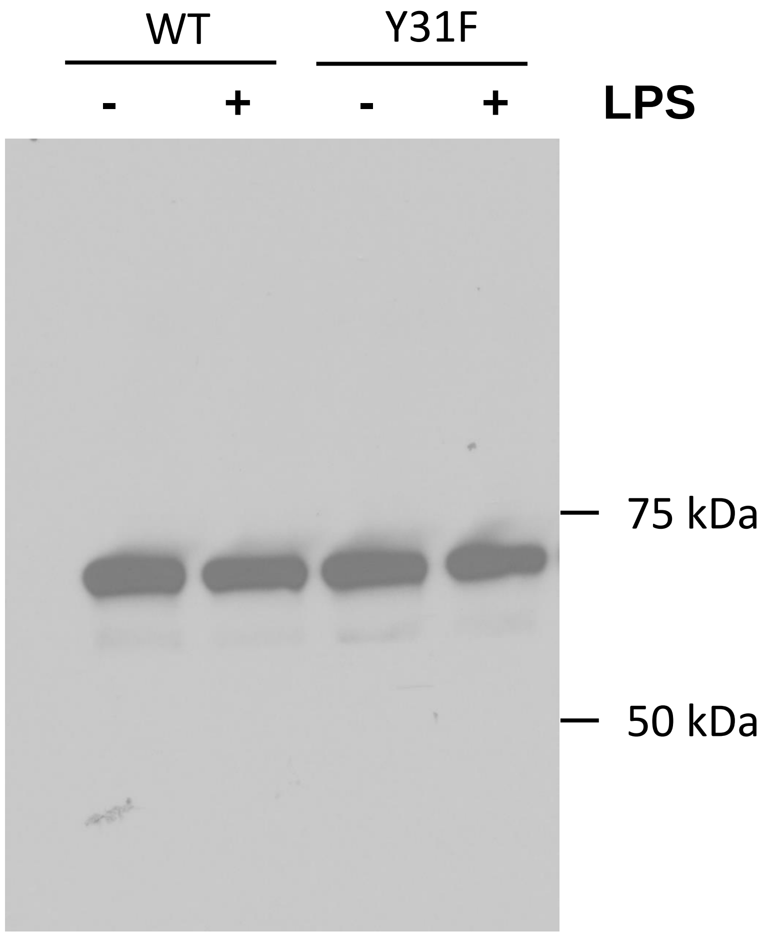

Fig 7A Pax

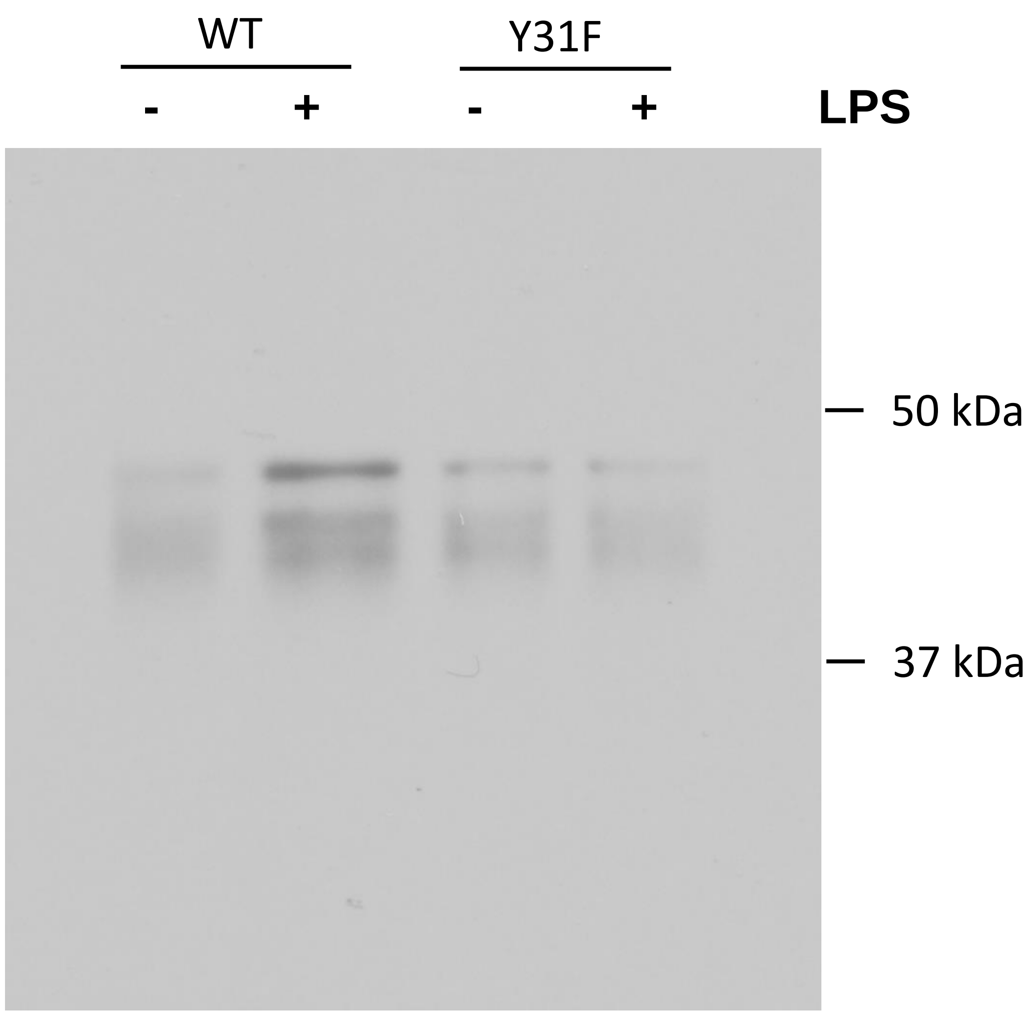

Fig7C Erk1/2

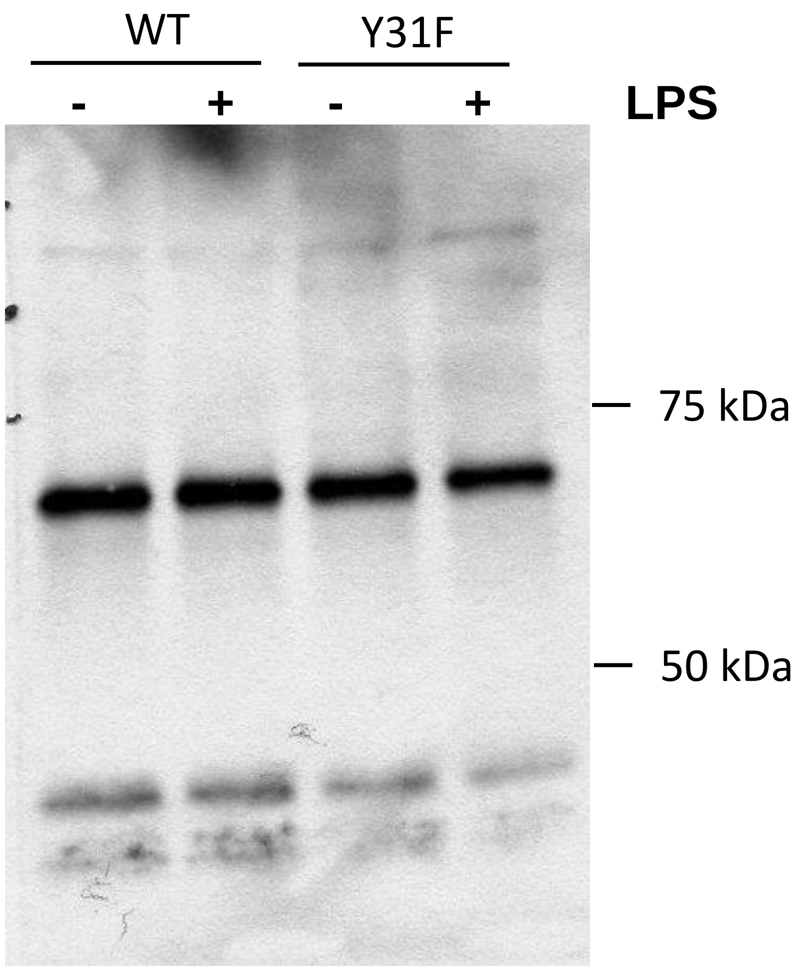

7C Paxillin

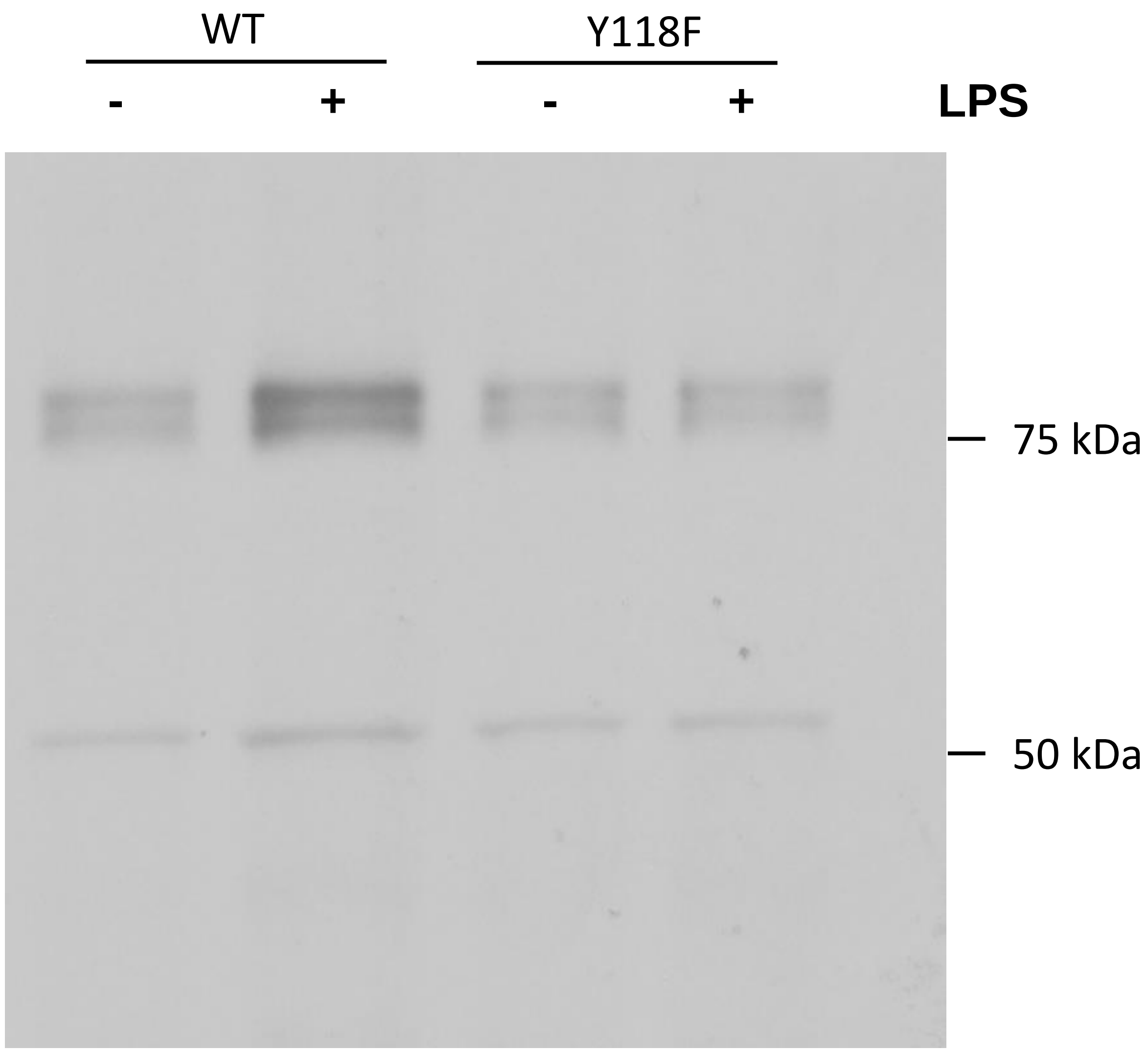

Fig7E Drp1

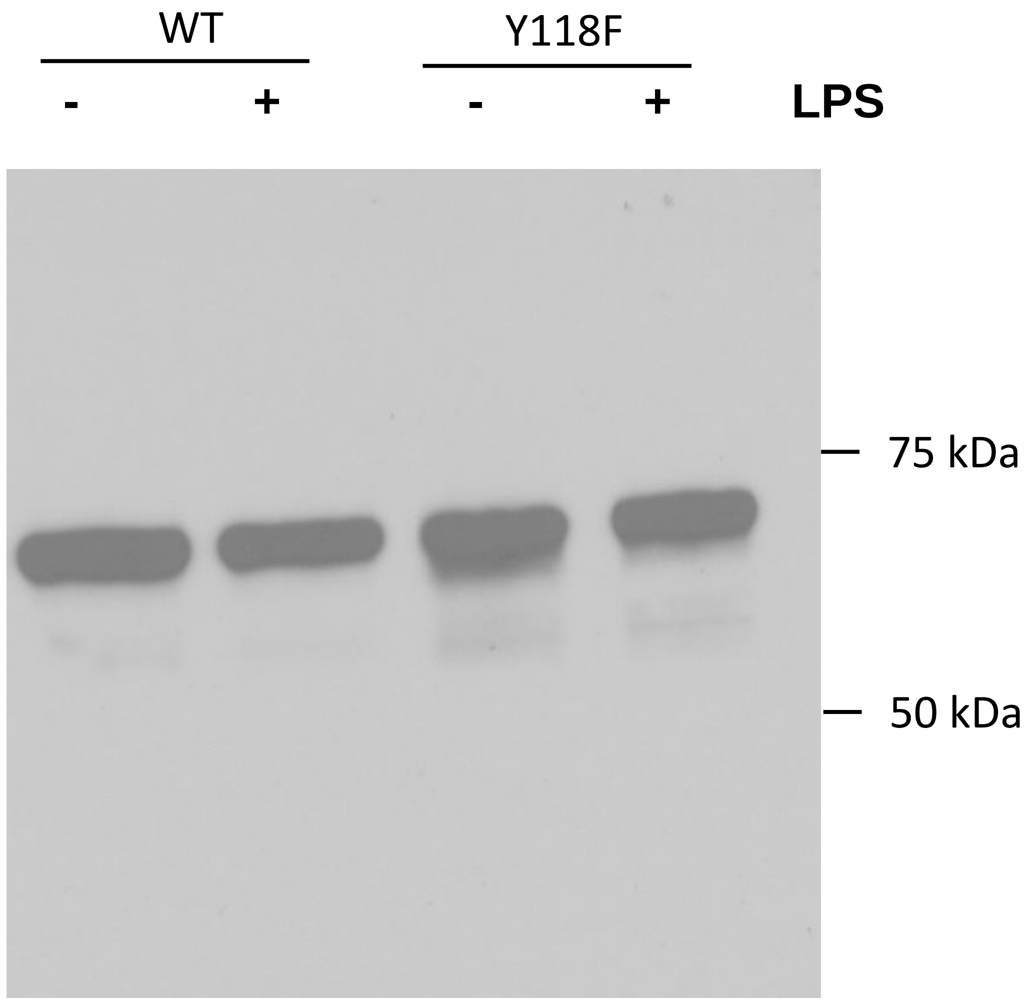

Fig7E Pax

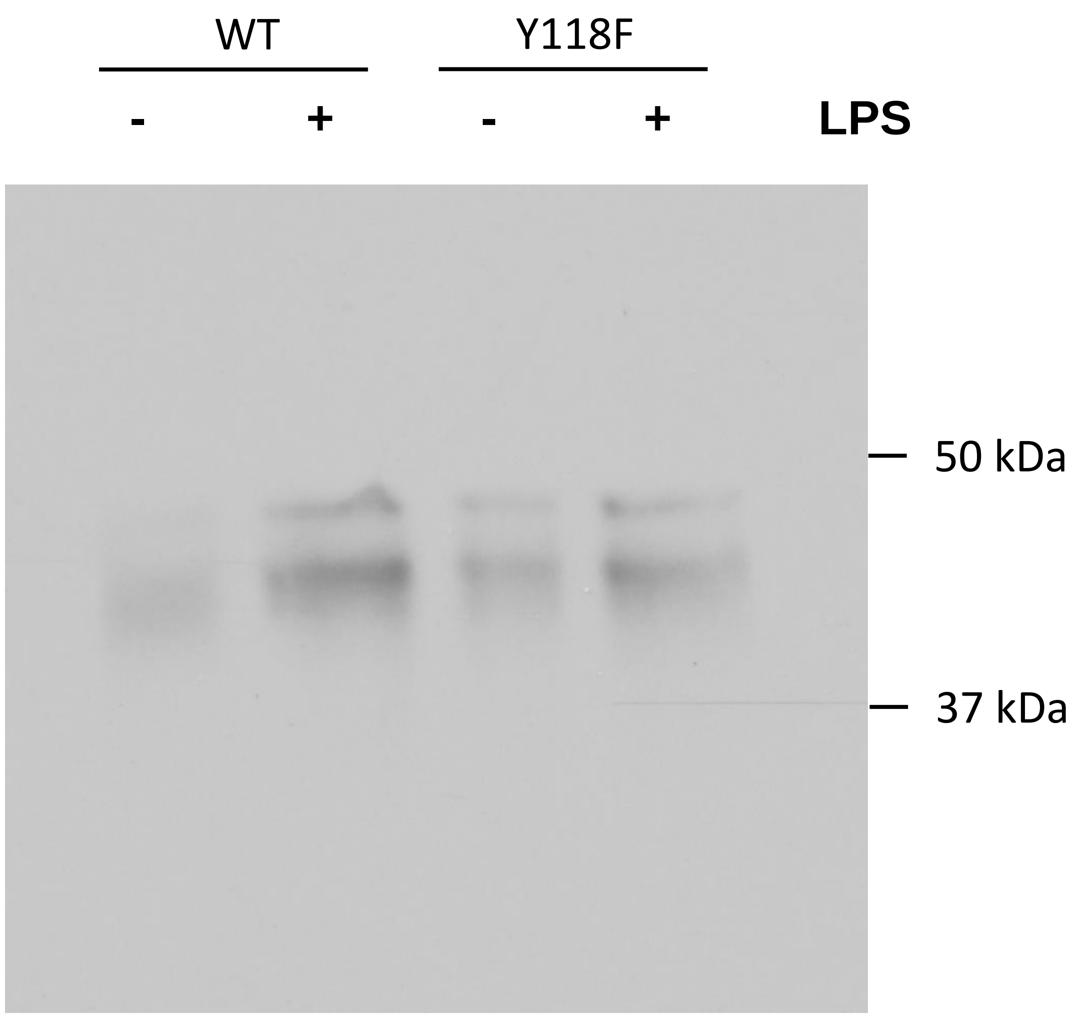

Fig 7G Erk 1/2

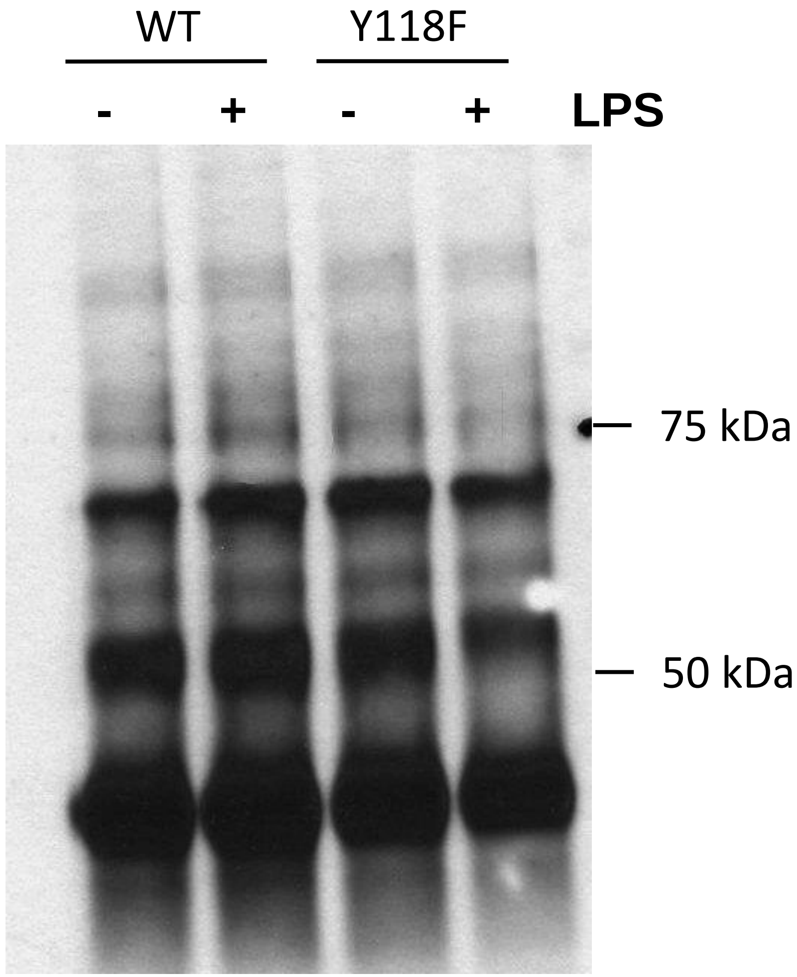

Fig 7G Paxillin

Fig 8A

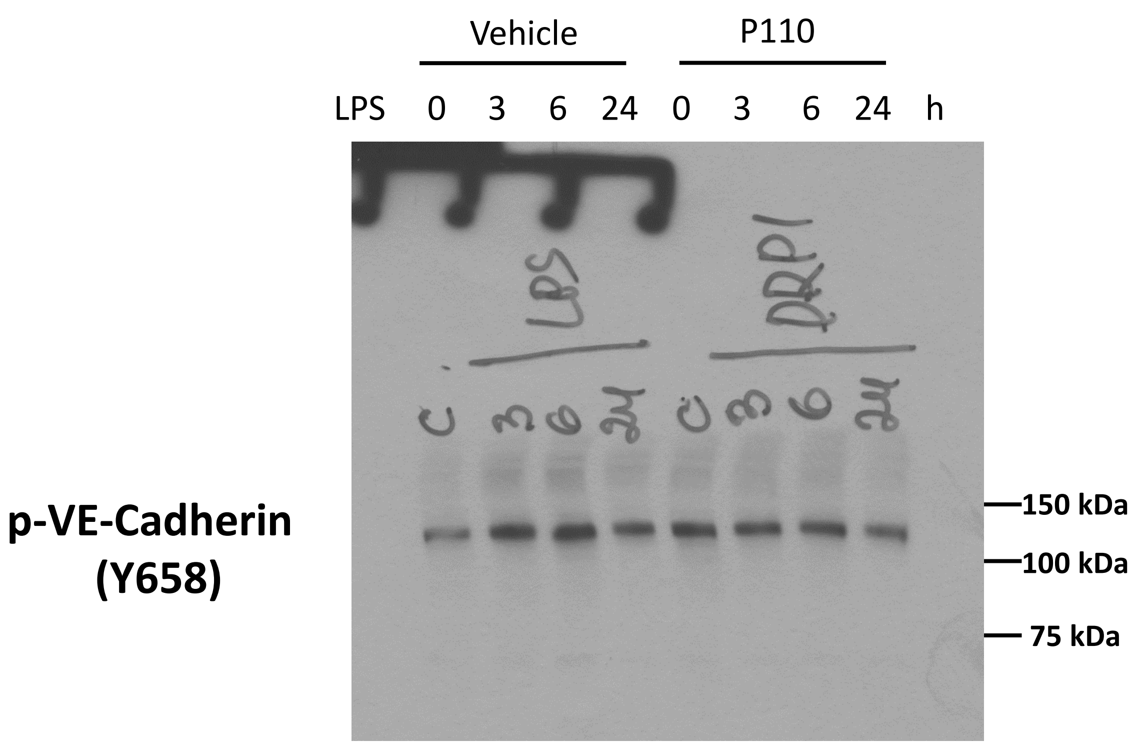

Vehicle
P110
LPS 0 3 6 24 0 3 6 24 h
p-VE-Cadherin (Y658)
150 kDa
100 kDa
75 kDa

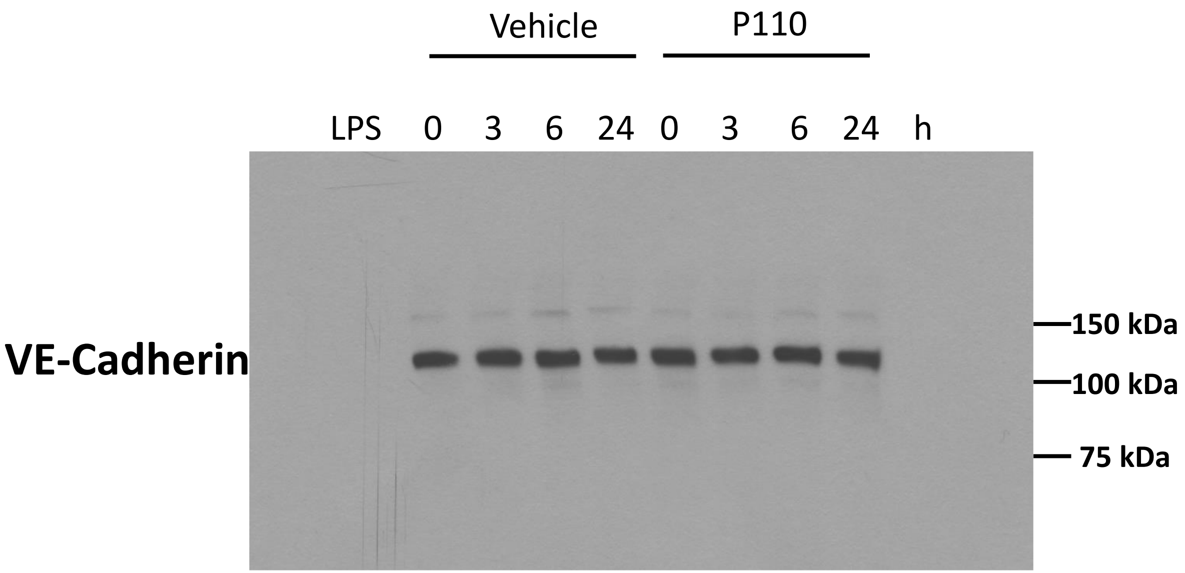

Vehicle
P110
LPS 0 3 6 24 0 3 6 24 h
VE-Cadherin
150 kDa
100 kDa
75 kDa

Fig 8C

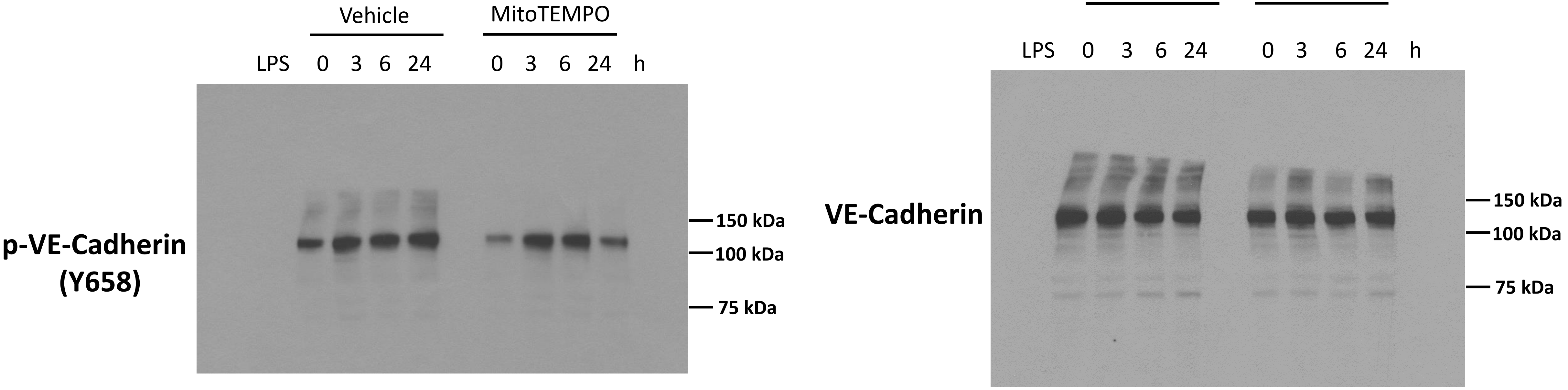
Vehicle
MitoTEMPO
LPS 0 3 6 24 0 3 6 24 h
p-VE-Cadherin (Y658)
150 kDa
100 kDa
75 kDa
Vehicle
MitoTEMPO
LPS 0 3 6 24 0 3 6 24 h
VE-Cadherin
150 kDa
100 kDa
75 kDa
